# Mitigation of AI adoption bias through an improved autonomous AI system for diabetic retinal disease

**DOI:** 10.1038/s41746-024-01389-x

**Published:** 2024-12-19

**Authors:** Michael D. Abràmoff, Philip T. Lavin, Julie R. Jakubowski, Barbara A. Blodi, Mia Keeys, Cara Joyce, James C. Folk

**Affiliations:** 1 Department of Ophthalmology and Visual Sciences, University of Iowa, Iowa City, IA USA; 2 Veterans Administration Medical Center, Iowa City, IA USA; 3 Digital Diagnostics, Inc., Coralville, IA USA; 4 Boston Biostatistics Research Foundation, Inc., Framingham, MA USA; 5 Baxter International Inc, Deerfield, IL USA; 6 Department of Ophthalmology and Visual Sciences, Wisconsin Reading Center, University of Wisconsin, Madison, WI USA; 7 Department of Public Health, George Washington University, Washington, DC USA; 8 Womens’ Commissioner, Washington, DC USA; 9 Department of Medicine, Stritch School of Medicine, Loyola University Chicago, Chicago, IL USA

**Keywords:** Machine learning, Diabetes, Diagnosis

## Abstract

Where adopted, Autonomous artificial Intelligence (AI) for Diabetic Retinal Disease (DRD) resolves longstanding racial, ethnic, and socioeconomic disparities, but AI adoption bias persists. This preregistered trial determined sensitivity and specificity of a previously FDA authorized AI, improved to compensate for lower contrast and smaller imaged area of a widely adopted, lower cost, handheld fundus camera (RetinaVue700, Baxter Healthcare, Deerfield, IL) to identify DRD in participants with diabetes without known DRD, in primary care. In 626 participants (1252 eyes) 50.8% male, 45.7% Hispanic, 17.3% Black, DRD prevalence was 29.0%, all prespecified non-inferiority endpoints were met and no racial, ethnic or sex bias was identified, against a Wisconsin Reading Center level I prognostic standard using widefield stereoscopic photography and macular Optical Coherence Tomography. Results suggest this improved autonomous AI system can mitigate AI adoption bias, while preserving safety and efficacy, potentially contributing to rapid scaling of health access equity. ClinicalTrials.gov NCT05808699 (3/29/2023).

## Introduction

A provocative publication by the Institution of Medicine, over 20 years ago, demonstrated substantial health disparities in the US healthcare system^1^. These disparities have remained an almost intractable problem, and scalable solutions are scarce^2,3^. There are multiple causes; in diabetes complications and especially diabetic retinal disease (DRD)^4^, lack of equitable access to early diagnosis and treatment^5–9^, are a major, though not singular source of such health inequity^10^. Randomized controlled trials (RCTs) and other studies have shown that autonomous Artificial Intelligence (AI) – making a medical decision without human oversight^11^ – for point-of-care, rapid DRD diagnosis improves access to the diabetic eye exam^12^, removes racial and ethnic access disparities^13^, and increases clinician productivity and satisfaction^14^, offering a scalable solution to a problem long considered intractable^15^. Such autonomous AI was originally De Novo authorized by FDA utilizing a desktop fundus camera, based on its safety and efficacy (LumineticsCore, Digital Diagnostics, Coralville, Iowa)^16^. The recent study on AI utilization by Wu et al.^17^, showed both rapid scaling – due to broad stakeholder support, sustainable reimbursement, and care gap closure^18,19^, - but also persistent *AI adoption bias* for this autonomous AI, as under-resourced clinics that serve racially minoritized, rural and low-income communities lag in adopting such technology^20^. Root-cause analysis through the recently published AI bias mitigation framework^21^, found that the cost, clinic space, and workflow burden of the above autonomous AI system, often exceeds the financial, staff expertise, and clinic space resources, particularly in under-resourced clinics^21^. Thus, utilizing an already widely adopted, lower cost, easier to use, one image per eye, handheld fundus camera optimized for underresourced clinicshas the potential to mitigate adoption bias, but requires safety and efficacy to be preserved.

The autonomous AI system was optimized for the lower contrast and smaller retinal area of such a camera *(rv700*; RetinaVue 700 Imager, Baxter Healthcare, Deerfield, IL, USA), by compensating for the reduced input image information through improved biomarker based diagnostic algorithms. A preregistered, Contract Research Organization (CRO; Fortrea Corp, Durham, NC) managed, intent-to-screen, non-inferiority study design was developed to evaluate this improved autonomous AI system, operated by minimally trained existing staff, in a representative sample of people with diabetes without diagnosed DRD. The aims of this study are to assess the safety, efficacy and access/adoption impact of the improved autonomous AI system.

## Results

### Study population characteristics

**Fig. 1:**
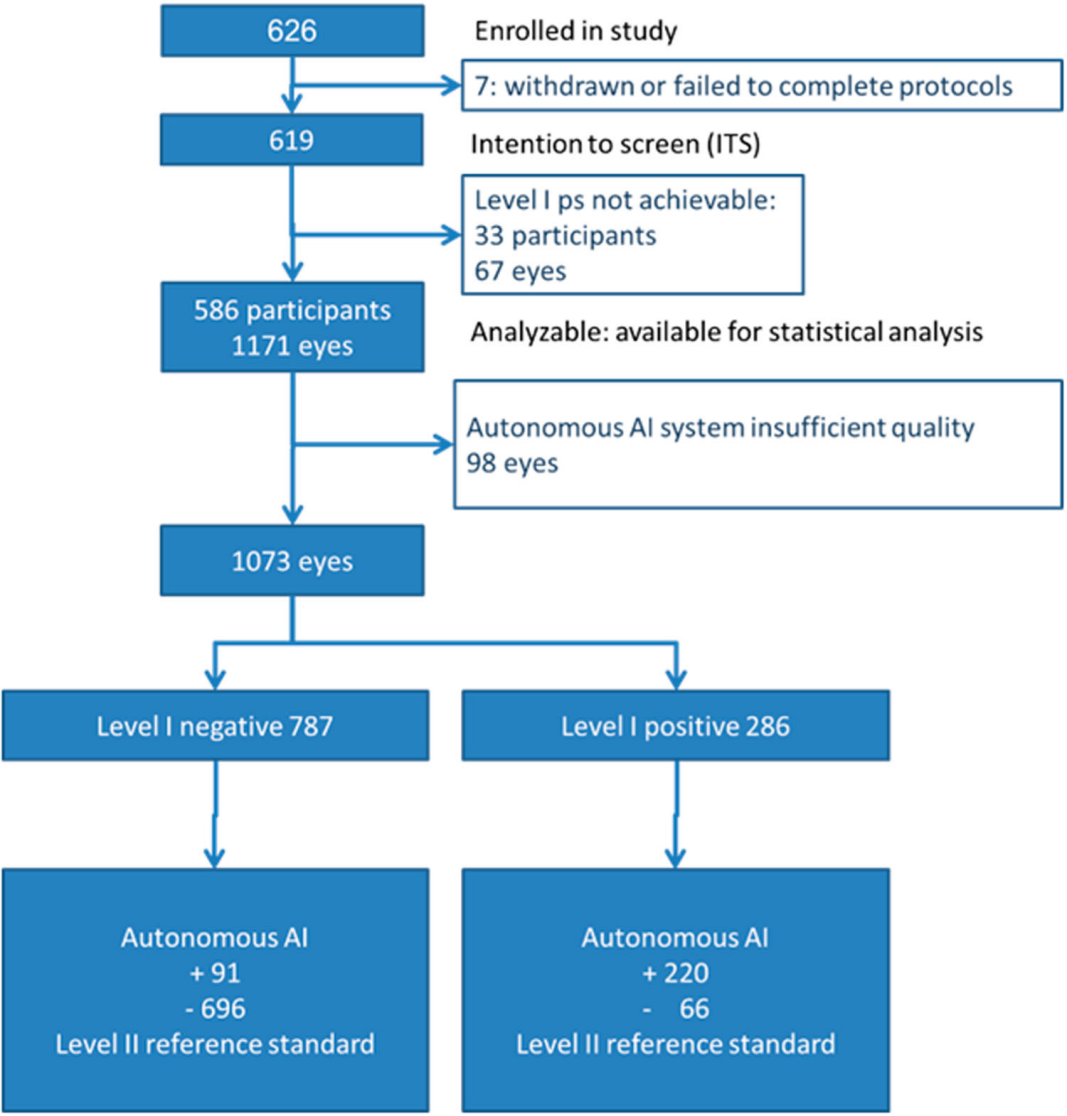
Waterfall diagram. Waterfall (STARD) diagram showing the final disposition of each participant in the enrolled, intention to screen (ITS), and fully analyzable populations.

A total of 626 participants (1252 eyes) were enrolled at 8 primary care sites, of which 619 (1238 eyes) completed all procedures. A subset of 567 (1073 eyes) of these participants could be fully analyzed, see Fig. 1 and Table 1. Prevalence of Early Treatment of Diabetic Retinopathy severity scale (ETDRS)^22^ > = 35 or Diabetic Macular Edema (DME) was 38.9% (221/567) among participants, and 29.0% (311/1073) among eyes; prevalence of vision threatening DRD (vtDRD) (ETDRS > = 53 or DME) was 5.6% (60/1073 eyes)^22^; see Table 2 for detailed prevalences; prevalence of DME was 4.0% (43/1073 eyes). Average centerfield thickness ± std was 243 µm (±26 µm): 245 µm (±35 µm) in the 221 eyes with ETDRS > = 35 or DME, and 242 µm (±22 µm) in those without. None of the participants had symptoms of vision loss, there were no adverse events.

**Table 1:** Demographics of participants and non-participants

	Analyzable (*N* = 567)	Not Analyzable (*N* = 52)	*P* value (2-sided)
Age (years) at Consent			0.0004
* n*	567	52	
Mean (SD)	54.1 (12.0)	60.3 (12.9)	
Median	55.0	60.5	
Min, Max	22.0, 87.0	26.0, 86.0	
Age Category			0.0011
<65 years	457 (80.6%)	31 (59.6%)	
65+	110 (19.4%)	21 (40.4%)	
Sex at birth, *n* (%)			0.5639
Male	288 (50.8%)	24 (46.2%)	
Female	279 (49.2%)	28 (53.8%)	
Ethnicity, *n* (%)			0.0653
Not Hispanic or Latino	304 (53.6%)	37 (71.2%)	
Hispanic or Latino	259 (45.7%)	15 (28.8%)	
Unknown or Not Reported	4 (0.7%)	0	
Race (all that apply), *n* (%)			0.4370
White	385 (67.9%)	33 (63.5%)	
Non-White	182 (32.1%)	19 (36.5%)	
American Indian or Alaska Native	13 (2.3%)	1 (1.9%)	
Asian	35 (6.2%)	1 (1.9%)	
Black or African American	98 (17.3%)	13 (25.0%)	
Latino	34 (6.0%)	5 (9.6%)	
Native Hawaiian or Other Pacific Islander	6 (1.1%)	0	
Refuse to provide	0	0	
Unknown	1 (0.2%)	0	
Other	2 (0.4%)	0	
Mixed Race	5 (0.9%)	1 (1.9%)	
HbA1c (%)			0.3234
*n*	558	52	
Mean (SD)	10.0 (1.99)	9.7 (2.24)	
Median	10.0	9.7	
Min, Max	4.6, 15.3	5.1, 14.4

**Table 2:** ETDRS level prevalence in the analyzable subset

ETDRS severity level	*n* (%)
10	545 (50.8)
12	95 (8.9)
14B	5 (0.5)
15	16 (1.5)
20	137 (12.8)
35 A	2 (0.2)
35B	12 (1.1)
35 C	57 (5.3)
35D	9 (0.8)
35E	27 (2.5)
35 F	106 (9.9)
43 A	17 (1.6)
43B	19 (1.8)
47 A	2 (0.2)
60	4 (0.4)
61 A	5 (0.5)
61B	9 (0.8)
65 A	1 (0.1)
65B	1 (0.1)
71 A	1 (0.1)
71 C	1 (0.1)
71D	1 (0.1)
90	1 (0.1)

### Autonomous AI system characteristics

At the eye level, preregistered sensitivity/specificity of the autonomous AI system against the Level II reference standard by the Wisconsin Reading Center (WRC) was 97.3% (one-sided 97.5% lower bound: 94.3%) and 82.7% (one-sided 97.5% lower bound: 80.0%), respectively, for detecting ETDRS > = 35 or DME. Preregistered sensitivity ${s}_{c}$ against the Level I reference standard, also by the WRC, was 79.6% (one-sided 97.5% lower bound: 75.1%), and specificity was 88.4% (one-sided 97.5% lower bound: 86.1%), both exceeding the non-inferiority endpoints (*p* = 0.021/*p* < 0.001), so that the null hypothesis could be rejected. Diagnosability was 90.6% (95% CI: 89.2%, 92.0%) at the eye level, and 15.5% of eyes needed pharmacologic dilation. At the participant-level, sensitivity against the Level I standard was 81.5% (one-sided 97.5% lower bound: 76.9%; *p* = 0.006) and specificity 82.2% (one-sided 97.5% lower bound: 78.4%; *p* = 0.008), respectively; diagnosability at the participant level was 95.8% (95% CI: 94.2%, 97.0%). There were no significant differences between racial, ethnic or sex subgroups, or at any intersections, for any of the above outcome parameters, see Table 3. See Table 4 for secomndary outcomes.

**Table 3:** AI bias: *p*-values for differences in eye-level sensitivity and specificity by sex, race, and ethnicity, all of which are non-significant; unadjusted for multiple comparisons, adjusting would make these even less significant

	Sex (Male vs female)	Race (Black vs non Black)	Ethnicity (Hispanic vs non-hispanic)
Sensitivity	0.066	1	0.090
Specificity	0.057	0.128	0.366

**Table 4:** Secondary outcomes

	Point estimate	Bounds
Positive Predictive Value (PPV)	70.6%	one-sided 97.5% lower bound: 64.1%
Negative Predictive Value (NPV)	87.9%	one-sided 97.5% lower bound: 83.9%
Positive Likelihood Ratio (PLR)	4.47	one-sided 97.5% lower bound: 3.00
Negative Likelihood Ratio (NLR)	0.26	one-sided 97.5% lower bound: 0.16

The improved autonomous AI system sensitivity against the level I reference standard was significantly higher, at 79.6%, than that of the WRC evaluating the same images, at 67.2%, *p* < 0.001 at the eye level (specificity 99.8%). WRC sensitivity failed the primary non-inferiority endpoint.

Among 60 vtDRD eyes, the improved autonomous AI system missed 14 cases (23%). Average centerfield thickness for these false negatives was 250 µm (±4 µm). One eye had ETDRS 60, one eye ETDRS level 61, and of the 12 false negative cases because of DME, centerfield thickness averaged 307 µm for both Center-involved DME (CIDME) and Clinically Significant DME (CSDME), none had ETDRS > = 20, none had symptoms of vision loss or thickness >360 µm. All of these false negatives were also missed by the WRC reading the same images (the Level II reference standard), and they missed 9 more eyes with vtDRD. A worst-case analysis was performed by assuming all (Level I) DRD eyes to be false negatives and all non-DRD to be false positives for those eyes receiving an insufficient image quality. Worst case analysis drops sensitivity to 64.5% (220/341), and specificity to 83.9% (696/830). Subjects with eyes determined by the AI as insufficient quality received a ‘referral to eye care provider’ output for patient safety.

In its pivotal trial, the ‘predicate’ autonomous AI, utilizing the higher cost, tabletop and harder to use, two image per eye, Topcon NW 400 (Topcon USA, Pyramus, NJ, USA) camera, was determined to have sensitivity ${s}_{c}=87.2 \%$, (95% CI, 81.8%–91.2%) at the participant level^16^. Using these and the present results gives a Population Achieved Sensitivity (PAS) PAS_NW400_/PAS_RV700_ threshold or break-even ratio of 1.07x (95% CI, 1.02–1.15).

## Discussion

The results of this preregistered, prespecified, Contract Research Organization (CRO) managed Good Clinical Practice (GCP)^23^ arms-length from the sponsor trial, confirmed the hypotheses of the safety, effectiveness and lack of in-equity of the improved autonomous AI system, designed to minimize AI adoption bias and thus maximize access to necessary health access equity. Sensitivity/specificity against the Level II reference standard by the WRC at the eye level to detect DRD (ETDRS severity scale 35 or higher or DME) was 97.3% and 82.7% with a diagnosability of 94.9%. It exceeded the non-inferiority endpoint at a sensitivity of 79.6% (*p* = 0.021), and specificity 88.4% (*p* < 0.001), at the eye level, against the Level I reference standard, in a sample representative of the US population with diabetes. At the participant level it also exceeded these non-inferiority endpoints, with sensitivity 81.5% (*p* = 0.006) and specificity 82.2% (*p* = 0.008). None of the outcomes showed evidence of racial, ethnic or sex biases in sensitivity or specificity.

**Fig. 2:**
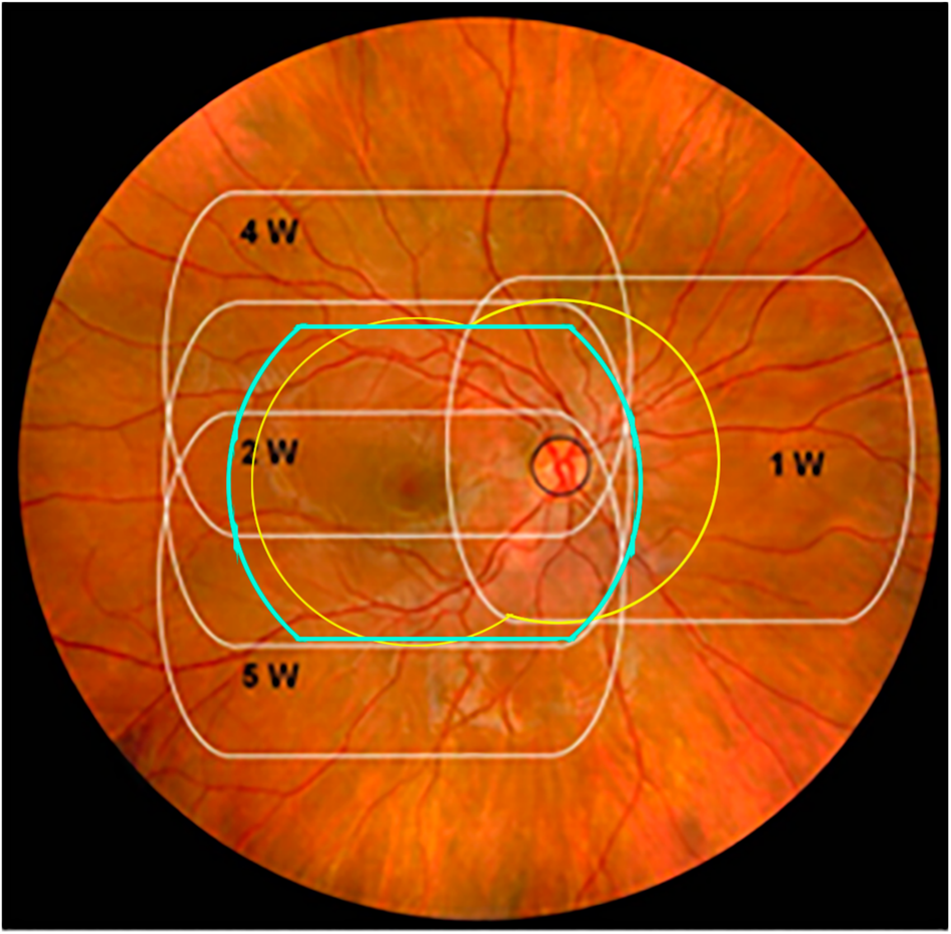
Retinal coverage of retinal cameras. Retinal areas of the posterior pole of the right eye covered by the ETDRS 4 widefield color stereo protocol, in white, the nw400 ‘predicate’ fundus camera two non-stereo image protocol, in yellow, and the rv700 low-cost, compact, handheld, easy-to-use one image per eye protocol, in blue, provided their respective imaging protocols are complied with. Any abnormality due to DRD that is not within the blue outline, but is within the white outlines, is, by definition, not available for the improved autonomous AI system that uses the rv700.

The sensitivity of the AI against the Level I reference standard, at 79.6%, was significantly (*p* < 0.001) higher than the 67.2% of the WRC reading the same rv700 images as the AI. The WRC has been considered the most established reading center in the world for DRD since 1979^24^, and has created the reference standard for >70% of all industry sponsored FDA intervention trials for DRD. While the improved autonomous AI system has lower sensitivity on the rv700 than the predicate on the nw400 images, measured against the Level I standard, the sensitivity of the WRC against this same Level I reference standard is significantly lower still. This is likely due to a camera effect, for which the AI was able to largely compensate - as it was designed to do - so the study endpoints were met. This camera effect is due to the rv700 imaging a smaller area of the retina, (Fig. 2) at lower image contrast^25^, reducing the amount of input information for the AI to make its diagnostic decision.

For the Level I prognostic standard, the WRC determines ETDRS severity levels, as well as the presence of DME, from high quality four widefield stereo color images (4 W) and macular optical coherence tomography (OCT) imaging, obtained by WRC certified ophthalmic photographers. For the Level II reference standard, ‘WRC reading of rv700 images‘, these same WRC readers grade only the rv700 images, while masked to the high quality images and OCT to determine DRD severity– thus, using the same retinal information that the AI system uses as input, see Fig. 2. While the improved autonomous AI system, using the rv700 images, met the non-inferiority endpoints, the WRC failed to meet them when presented with exactly the same input image information. The results demonstrate that autonomous AI has higher sensitivity on the rv700 for early diagnosis of DRD, as it is well established that individual graders evaluating retinal images, including rv700 images in a telemedicine setting, do not approach the sensitivity performance of the WRC – which didn’t meet this endpoint^26,27^.

The sensitivity of the improved autonomous AI system is 81.5% against the Level I standard at the participant level: while high enough to exceed the non-inferiority endpoints, this is lower than the 87.2% sensitivity of the predicate using the nw400 camera against the Level I standard. This tradeoff results from the Total Product Lifecycle-Bias Mitigation (TPLC-BM) analysis^21^, in order to mitigate the AI adoption bias that was identified^17^. PAS for these two autonomous AI systems has a break-even ratio of 1.07x (95% CI: 1.02–1.15), meaning that, if adoption of the improved AI system is at least 1.07x higher than of the predicate AI, *more* true patients with DRD will be identified with the improved AI than with the predicate. Many hundreds of the predicate autonomous AI system (using the nw400) have been adopted since FDA authorization in 2018, making it the fastest growing medical AI in terms of patient utilization based on claims data^17^. Since 2020, many thousands of rv700 cameras have been adopted, albeit in a telemedicine setting using human readers. Thus, potential widespread adoption of the improved autonomous AI can be expected to result in a PAS that is also an order of magnitude larger, substantially over the 1.07x break-even PAS ratio, given comparablze diagnosability. We used the lower diagnosability found in this study for the rv700 PAS, even though dilation was used in 15.5% of subjects compared to 23.6% in the predicate autonomous AI pivotal trial^16^, with the nw400, to bias the analysis against the improved autonomous AI system. Other studies have found dilation rates as high as 40%. Consequently, a rapid positive impact at the population level can be expected because *more* patients that have DRD and can benefit from treatment or close management will be identified with the adopted improved AI system, than with the predicate^21,28^, scaling health access equity, through point of care diagnosis which allows for timely referral and counseling.

As mentioned in the Introduction, RCTs have already established that the use of autonomous AI for the diabetic eye exam reduces health disparities and improves health access equity^12,13^. Improving adoption by reducing AI adoption bias is the next frontier^17^, so it is crucial that when cleared for clinical usage, post-market continuous efficacy monitoring conforms to the TPLC-BM, to determine whether adoption bias is indeed being mitigated^21^. Given the focus on AI bias that this autonomous AI system is designed to address, it is important that new sources of AI bias are not introduced. The design, development and validation was performed per the TPLC-BM framework for AI^21^, additionally the results showed no significant racial, ethnic or sex bias was present in sensitivity and specificity.

The autonomous AI system missed cases of DRD, including 14/60 eyes with vision threatening DRD; all of these were also missed by the ‘WRC reading rv700 images’, primarily because the lesions in these eyes were outside the area of the retina imaged by the rv700, see Fig. 2. Clinically, none of these 14 eyes had symptoms of vision loss, their centerfield thickness averaged 277 µm, one eye had ETDRS 60 (status after panretinal photocoagulation), one eye ETDRS level 61, and all of the others were ETDRS < = 20. Thus, none of these eyes qualified for immediate treatment with anti-vascular endothelial growth factor, steroids or other^29,30^.

The results show the safety and efficacy, as well as lack of racial and ethnic inequity of the improved autonomous AI. WRC experts show significantly lower sensitivity, compared to the improved autonomous AI, using the level I reference standard. Scientific and professional societies recommend in the chair indirect ophthalmoscopy and biomicroscopy, performed by ophthalmologists and retina specialists^30,31^. Sensitivity for this standard practice is even lower, around 30–40%, using the level I standard, according to the two comparison studies available in the literature on clinician accuracy^27^.

According to the largest study to date, using claims data, only 15.3%^32^ of people with diabetes get a regular diabetic eye exam. While sensitivity of the improved autonomous AI is slightly lower than that of the predicate autonomous AI, it is significantly more sensitive than either the current standard practice of teleretinal imaging or clinical exams by ophthalmologic clinicians. However, this preferred practice has not succeeded in addressing either the substantial health inequities, nor expanding access, as explained in the Introduction. In contrast, the improved autonomous AI system was explicitly designed to vastly expand access to diabetic eye exams specifically in underserved communities.

The results reinforce the importance of the choice of reference standard against which any AI is compared, as expressed by the metric for reference standard quality^28^. If the reading center based Level II is used instead of the most rigorous, Level I prognostic standard, the sensitivity of the AI seemingly improves from 79.6 to 97.3%, at the eye level. Obviously, its true performance did not change –these apparent differences are caused by the difference in reference standard: where the Level II standard uses the same images as the AI system, the Level I standard is based on a much larger retinal area imaged at high contrast in stereo as well as OCT performed by highly experienced WRC certified ophthalmic photographers, Fig. 2. The Level I prognostic standard is directly tied to what patients and their providers care about – clinical (visual) outcome^28,33^. Still, most image based medical AI – in any specialty – continues to be validated against Level II or even Level III (derived from multiple clinical experts, not part of a formal reading center) reference standards, and rarely are they compared against a prognostic standard as in the case of the autonomous AI for the diabetic eye exam, making valid comparisons challenging.

The results show the importance of developing (autonomous) AI under an ethical framework^33,34^, as the resulting metrics developed with FDA and other healthcare stakeholders^21,28^ allowed careful quantitative analysis of both individual benefit as well as health equity impact. This allows a balance where the autonomous AI is both safe and effective under criteria previously established (sensitivity and specificity meeting independently established non-inferiority endpoints)^16^, and at the same time maximizes the health equity impact, as quantified through PAS^21,28,35^.

The results also demonstrate how the autonomous AI algorithm output is tied to clinical outcome, if the patient is never treated. An autonomous AI output of “disease present”, i.e., ETDRS > = 35 or DME present, confers a ~18.5% risk of that patient having proliferative or worse DRD within 3 years, or a risk of ~11% of moderate or worse vision loss within 1 year, and ~35% in 3 years, if the patient were not treated. A “disease present” output thus maps to International Classification of Diseases (ICD)-10 category E11.339x: “Type 2 diabetes mellitus with moderate diabetic retinopathy without macular edema”, for the appropriate (“x”) laterality for a type 2 diabetes patient, as all patients will have at least this level of disease; while some patients with a “disease present” output will have biomarkers corresponding to more severe ETDRS levels or to DME, they will all have the E11.339x level of disease. A ‘disease not present’ output confers a risk for any of these outcomes below 1.8%. DRD terminology is often confusing, hence the current project to create a novel grading system for DRD^36^. For example, under the International Classification of Diabetic Retinopathy, ETDRS 35 is termed *moderate*^37^, but under ETDRS itself, it is described as *mild*^22^. We strictly use the ETDRS terminology where possible, rather than using the terms ‘mild’ or ‘moderate’, as they tend to introduce confusion.

A limitation of this study is that it was not intended or designed to determine whether the improved autonomous AI system improves health equity. It was designed to determine safety, efficacy and lack of in-equity of the improved autonomous AI. It had a sufficient number of cases and controls to test the hypotheses and confirm safety, efficacy; no inequity signal was found (no undesirable ethnic or racial bias). However, previous RCTs and retrospective studies^12,13^ of the predicate autonomous AI with the nw400 showed improved real world health equity (i.e., it reduced racial and ethnic disparities). Such future real world studies will have to be performed also for the improved autonomous AI system once FDA authorized.

Key in AI validation trials is that the sample and workflow are representative of the population the AI will be used in, after FDA clearance, as underlined by our work with US FDA on this subject^21,28^. Omitting such constraints introduces impossible to correct for bias and overestimation of accuracy and patient benefit, resulting in substantial patient risk and poorer outcomes, as shown in the Fenton, et al*.* study^38,28^. For example, some validation studies of other autonomous AI have included subjects in clinical care for DRD to enrich the sample. However, this biases the sample in favor of those DRD phenotypes that are easier to diagnose by clinicians, and against those where the true state of disease has historically been hard to determine by clinicians, such as venous beading in 2 quadrants exclusively, a well known marker for ETDRS 53^39^.

The prevalence of ETDRS > = 35/DME in this study at the subject level was comparable to other recent primary care based studies at around 20-25%, though prevalence can vary based on how long a DRD screening has been deployed. While less recent studies from around the world showed higher prevalence^40^, these recent studies in the intended use environment show that estimates from this study are likely to reflect performance in the real world^13,41^.

In conclusion, this preregistered arms-length trial showed that the improved autonomous AI system utilizing a widely adopted, lower cost, easier to use, handheld camera to minimize AI adoption bias and designed to compensate for the lower image quality, retains safety and efficacy. It thereby has the potential to maximize health equity, as adoption bias in under-resourced clinics can be minimized because of the handheld, compact, lower cost, and easier to use one image per eye camera. At an increased adoption of at least 1.07x – and rv700 has already been adopted an order of magnitude more than the predicate - population achieved sensitivity PAS will increase, so that *more* patients with DRD in a given diabetes population will be identified than with the predicate, while retaining diagnostic accuracy^21,28^. These are the patients that will benefit from earlier management and treatment of DRD and their diabetes. In fact, the improved AI system outperforms even the most experienced retinal experts reading the same images. RCTs and other studies have established that autonomous AI can reduce health disparities in under-resourced clinics serving minority, rural and low-income populations, but AI adoption bias remains a major hurdle. The improved autonomous AI system is designed to mitigate this pernicious form of AI bias, and has the potential to increase adoption by under-resourced clinics in order to reach better visual outcomes, health equity and access to care for all people with diabetes.

## Methods

### Study design

From March 3, 2023 to November 30, 2023, participants were prospectively enrolled in this preregistered observational study at 8 primary care practice sites throughout the United States. The study protocol was approved by the Institutional Review Board (Advarra Inc, Columbia, MD 21044), for each site, (Approval # Pro00061789), all participants provided written informed consent and adhered to the Declaration of Helsinki. The study, which was funded by Digital Diagnostics Inc, was designed by the authors with input from the U.S. Food and Drug Administration (FDA) on the endpoints, statistical testing, and study design. The study protocol, endpoints, primary and secondary outcomes, and their statistical analysis and hypothesis testing were preregistered on March 29.2023 on ClinicalTrials.gov ID NCT05808699.

### Autonomous AI diagnostic system

The improved autonomous AI system, (LumineticsGo, Digital Diagnostics Inc, Coralville, Iowa), is paired with the RetinaVue 700 Imager (rv700, Baxter, Deerfield, IL), handheld portable fundus camera, and has two core AI components;

1. rv700: a lower cost, compact, handheld, fundus camera, allowing one image per eye, with reduced image contrast, covering less retinal area than the ‘predicate’ 2 image per eye NW400 protocol, and substantially less retinal area than the ETDRS 4 W imaging protocol, as in Fig. 2.
2. Assistive AI for image quality: essentially the same image quality system as in the original system^16^, which is implemented as multiple independent detectors for retinal area validation as well as focus, color balance and exposure, and has been modified to support one image per eye and lower image contrast. It is used assistively by the operator to detect, in real time, sufficient image quality or not, and thereby recommend whether an image should be retaken.
3. Autonomous diagnostic AI, which is based on the original autonomous AI^16^, and has been studied extensively over two decades^42–44^. It consists of multiple, partially redundant (statistically partially dependent) validated detectors for biomarkers, including hemorrhages, neovacularizations, exudates, and other lesions characteristic for DRD in the form of multilayer convolutional neural networks (CNN)^45,46^, and has been modified to support a lower cost, compact, handheld one image per eye camera, while retaining at least the same performance on the predicate fundus camera two image protocol.

The autonomous AI algorithms are ‘physiologically plausible’ to a limited degree due to their multiple, redundant, lesion-specific detectors for biomarkers^47^. Such detector based AI systems have multiple advantages over straight shot image based CNN AI: increased robustness against small perturbations in input images^48^, racially and ethnically invariant to retinal pigmentation, as per FDA’s approach^16,21,44^, and lower computational complexity, -less than 10 ^26^ floating point operations - urged by the recent US White House Executive Order on AI^49,50^.

The complete AI system was locked before the start of this study and placed in escrow at the Algorithm Integrity Provider.

### Study population

The target population was asymptomatic persons, ages of 22 and older, who had been diagnosed with diabetes and had not been previously diagnosed with DRD. A diagnosis of diabetes was defined as in all our studies^16^, meeting the criteria established by either the World Health Organization (WHO) or the American Diabetes Association (ADA); Hemoglobin A1c (HbA1c) ≥ 6.5% based on repeated assessments; Fasting Plasma Glucose (FPG) ≥ 126 mg/dL (7.0 mmol/L) based on repeated assessments; Oral Glucose Tolerance Test (OGTT) with two-hour plasma glucose (2-hr PG) ≥ 200 mg/dL (11.1 mmol/L) using the equivalent of an oral 75 g anhydrous glucose dose dissolved in water; or symptoms of hyperglycemia or hyperglycemic crisis with a random plasma glucose (RPG) ≥ 200 mg/dL (11.1 mmol/L)^51,52^. Exclusion criteria are listed in Supplemental Table S1 and includes any persistent vision loss, blurred vision that cannot be corrected, or floaters.

### Study and site initiation

Fortrea, a CRO, provided overall site and project management, including data management and independent monitoring services for all sites, as well as interdicting access to these by the Sponsor. The CRO was responsible for ensuring all sites adhere to GCP^23^ and comply with applicable guidelines for study execution. Fortea acted as Algorithm Integrity Provider (AIP), contracted to lock the AI system, hold any intermediate and final results and images in escrow, and interdict access to these by the Sponsor, from prior to the start of the study until final data lock. Boston Biostatistics Research Foundation conducted all analyses. Because the Sponsor was interdicted from access to the participants or AI system, the AIP performed all necessary maintenance and servicing activities during the study as well as throughout closeout. To ensure scientific rigor, the study, including Statistical Analysis Plan, was registered before study start at ClinicalTrials.gov under NCT05808699. See Supplementary materials for the preregistered protocol and statistical analysis.

All primary care sites in the study identified one or more in-house operator trainees to perform the *AI system protocol* (see below). After installation of the equipment by the Sponsor at the site, but before any participant was recruited, AI system operator trainees had to attest that they had not previously performed ocular imaging. Also, before start of study recruitment at each site, AI system operator trainees underwent a one-time standardized 2 h training program. They were trained how to acquire images, how to improve image quality if the AI system gave an insufficient quality output, and how to put images for analysis into the AI system. No additional training was provided to any of the AI system operators for the duration of the study. Independently, WRC certified expert photographers were identified in geographic locations close to each site by the CRO, and documented 4 W WRC certification was required before any participant was imaged^53^. The CRO completed site initiation visits at each site to ensure each site met all the GCP requirements prior to start of enrollment.

### Study protocol

All participants consented to participate in both the *AI system protocol* as well as the *WRC imaging protocol*, using two different cameras:

The *AI system protocol* consisted of the following steps:

1. operator takes images with the rv700 according to a standardized imaging protocol (Fig. 2);
2. operator submits images to the autonomous AI system for automated image quality and protocol adherence evaluation;
3. if the AI system outputs *insufficient quality*, steps 1–2 are repeated until *sufficient quality* is output or 3 attempts were made. If the AI system still indicates that images are of insufficient quality, the participant’s pupils are dilated with tropicamide 1.0% eyedrops, until the pupil diameter is at least 5 mm in each eye or 30 min have passed, and steps 1–2 are repeated until *sufficient quality* is output or 3 attempts were made. If the AI system still outputs that images are of insufficient quality, the AI system output of insufficient quality is automatically provided to the CRO via secure data transfer;
4. whenever the AI system indicates sufficient quality, the AI system disease level output (either *ETDRS* > = *35 or DME detected* or *not detected*) is automatically provided to the CRO via secure data transfer;

The final AI system output provided to the CRO after this protocol was either *ETDRS* > = *35 or DME detected*; or *ETDRS* < *35 and DME not detected;* or *insufficient quality*

The WRC imaging protocol was then conducted, always after pharmacologic dilation, and consisted of the following steps, all performed by a WRC certified photographer:

1. if participant is not already dilated, tropicamide 1.0% dilating eye drops are administered;
2. digital widefield stereoscopic fundus photography is performed, using a camera capable of widefield photography (Maestro, Topcon Medical Systems, Oakland, NJ) according to the WRC 4 W stereo protocol, by a WRC certified photographer^53^;
3. anterior segment photography for media opacity assessment is performed according to the Age Related Eye Disease Study^54^, by a WRC certified photographer;
4. OCT of the macula is performed using a standard OCT system capable of producing a cube scan containing at least 121 B scans, (Maestro, Topcon Medical Systems, Oakland, NJ) according to the WRC OCT protocol, by a WRC certified photographer^53^.

The WRC certified photographers were masked to the AI system outputs at all times. After completion of the imaging procedures, the CRO transferred all images (including the RV700 images) to the WRC.

### Reference standards and clinical outcome

Two Reference Standards were created based on the images collected: a prognostic standard, i.e., the highest level I reference standard, required to have a known relationship with clinical outcome, and a level II reference standard, determined by a validated reading center, but where the relationship to outcome has not been decisively determined^28^. To determine the Level I Prognostic Standard, the 4 W and macular OCT images were graded by three WRC retinal grading experts who independently graded each image according to the ETDRS and DRCR severity scales, using a majority voting paradigm^22,55,56^. CSDME was identified from 4 W if there was either retinal thickening or adjacent hard exudates <600 µm from the foveal center, or a zone of retinal thickening >1 disc area, part of which is less than 1 disc diameter from the foveal center, according to the WRC, in any eye^22,53,57^. CIDME was identified, from the macular OCT images, according to the DRCR grading paradigm^29^, if a participant had central subfield (a 1.0 mm circle centered on the fovea) thickness that was >300 µm, in that eye^58^. Because the prognostic standards for ETDRS, CIDME and CSDME have been linked to visual outcome, the risk of moderate or more vision loss given a “ETDRS > = 35 or CIDME or CSDME present” output from the autonomous AI can be determined, as follows^59^.

For ETDRS > = 35, the risk of Proliferative Diabetic Retinopathy at 3 years, based on observational studies^22,60,61^ and the ETDRS RCT with an arm that left patients untreated, was 18.5%, whereas ETDRS < = 20 conferred a risk <= 1.8%. For CSDME, the last RCT that had an arm which left participants with CSDME untreated, according to the ETDRS imaging and laser protocol, showed that the risk of moderate or worse vision loss (15 or more letters loss on the standardized ETDRS chart) for control arm participants with CSDME+ at 1 years was 8% and 24% after 3 years^62^. Without CSDME and ETDRS < 20 the risk was ~1.4% at both 1 year and 3 years. For CIDME, there has been no untreated arm in any RCT. The outcomes of the last RCTs, RISE and RIDE for ranibizumab for CIDME^63^, which had a laser photocoagulation arm (which in turn formed the treatment arm in the ETDRS RCT) showed that the risk in the photocoagulation arm was 5% at 1 year and 12% at 3 years for CIDME. Extrapolation by combining a weighted combination of the CSDME treated and CIDME photocoagulation risks leads to CIDME+ having a risk of moderate or worse vision loss of ~11% at 1 year and ~35% at 3 years^63^. CIDME and CSDME were combined into a DME present (or not) label for level I, and vision threatening DRD (vtDRD) was defined as ETDRS > = 53 and/or DME.

The second Reference Standard was a Level II Reference Standard, i.e., determined by a validated reading center (the same WRC readers), but this time instead of using 4 W and OCT, using the same information the AI uses to make its diagnosis, i.e., only the rv700 images, one per eye, according to the ICDR severity scale, and masked to 4 W and OCT, as well as masked to the Level I readings. The Level I Prognostic Standard require 4 W and OCT images obtained by certified ophthalmic photographers, under dilation. rv700 images are neither stereo, nor widefield, only one field per eye, and obtained by minimally trained operators rather than certified ophthalmic photographers, and were not part of the original ETDRS trial. As such only a reference standard level II can be determined. The Level II reference standard thus allows any decrease in performance due to less retinal area and lower image contrast, see Fig. 2, to be isolated, because both expert readers that create the Level II standard and the diagnostic AI algorithms have exactly the same input image information to base their output on.

The rationale for the ‘ETDRS > = 35 or DME’ cut-off is as follows: it follows the American Academy of Ophthalmology (AAO) preferred practice pattern^30^, where only those patients with any eye up to ETDRS 20, i.e., less than ETDRS 35, are recommended to be seen at 12 months interval. With any eye at ETDRS > = 35 the recommended interval is shorter, because the risk of poor outcome at that level and up is much higher, as analyzed and documented above; this also conforms to the 2018 FDA De Novo clearance for the predicate autonomous AI^16^.

WRC staff, primary care site personnel, Sponsor personnel, and the statistical team were masked at all times to the AI system diagnostic outputs.

### Outcome parameters

Primary outcomes were sensitivity and specificity of the autonomous AI system against the Level I prognostic standard at the eye-level. Secondary outcomes are sensitivity and specificity against the Level II reference standard at the eye and participant level, sensitivity and specificity at the participant-level, diagnosability at the eye and participant level, sensitivity and specificity of the Level II reference standard against the Level I prognostic standard, sensitivity and specificity without bootstrapping, positive predictive value (PPV) and negative predictive value (NPV), positive and negative Likelihood Ratio (PLR and NLR), and sensitivity and specificity that impute “worst-case” scenario values. Post-hoc analysis (i.e., not preregistered) included Population Achieved Sensitivity (PAS) and PAS ratio threshold.

The thresholds for FDA clearance were 80% for sensitivity, 80% for specificity^16^ at the subject level, established through an extensive FDA led Delphi process using clinical experts from around the world, as described in Abramoff et al.^28^. As AI focusesx more on per eye level, those thresholds were transposed to the eye level. Sample sizes of 200 eyes with Early Treatment of Diabetic Retinopathy (ETDRS) severity scale $\ge 35$ and/or DME, including at least 20 eyes with ETDRS > = 53 to mitigate spectrum (disease severity) bias, and 140 eyes with ETDRS < = 20 and no DME were determined to be sufficient, and able to rule out sensitivity and specificity inferiority thresholds (with one-sided 97.5% confidence bound) to reflect non-inferiority margins (5% for sensitivity, 2.5% for specificity). Sample sizes were chosen to provide adequate power for the null hypotheses for sensitivity and specificity. Additionally, both Lundeen et al.^64^, as well as the pivotal trial of the original autonomous AI^16^, established that approximately 20% of ‘ETDRS > = 35 or DME’ eyes are ‘ETDRS > = 53 or DME’, prompting our inclusion of an additional minimum acceptable sample size within this clinically important stratum. The CRO received all final WRC gradings and the final AI system outputs for all eyes. There were no interim analyses. The analysis was conducted following statistical analysis plan finalization and final database lock.

PAS and their ratios were prespecified, as developed in the work by Abramoff with FDA^21,28^. PAS measures the number of patients identified that truly have the disease in a given population, and quantifies the effects of adoption bias:

1 $${PAS}=\frac{{s}_{c}c{p}_{c}{d}_{c}\,}{c{p}_{c}+\left(1-c\right)\hat{{p}_{{nc}}}}\cong {s}_{c}c{d}_{c}$$

with:

*s*_*c*_ = *sensitivity*

*d*_*c*_ = *diagnosability*

*c* = *access*

*p*_*c*_ = *measured prevalence in the subpopulation with access*

$${\hat{{p}}}_{{nc}}={estimated\; prevalence},{in\; the\; subpopulation\; without\; access}$$

We conservatively assume prevalence *p* will be the same in the subpopulation lacking access *c* as in the subpopulation that has access – depending on the causes, it is likely that *p* is larger in the subpopulation without access. We have conservatively used *p* = 0.2 for both subpopulations, based on recent real world studies^41^. Because *c* is hard to determine for new technology with yet limited adoption, we eliminate *c* by calculating the *ratio* of PAS_nw400_/PAS_RVrv700_. This ratio expresses the threshold at which adoption of the improved autonomous AI (in this study, with the rv700) results in equal numbers of at risk patients identified in a given population compared to the predicate (with the nw400), even though sensitivities differ. Above this break-even ratio, the improved autonomous AI system will identify more patients with DRD in a given population than the predicate. The sensitivity ${s}_{c}$ (participant level) and diagnosability ${d}_{c}$ for the predicate autonomous AI (with the nw400) is taken from its pivotal trial, the sensitivity ${s}_{c}$ and diagnosability ${d}_{c}$ for the improved autonomous AI (with rv700) from the present results.

### Statistical analysis

Study success was pre-defined as both sensitivity and specificity of the autonomous AI system, and the hypothesis of interest was

2 $${H}_{0}:p \,<\, {p}_{0}{\rm{vs}}.\,{H}_{A}:p\ge {p}_{0}$$

To preserve Type I error, study success was defined as requiring both null hypotheses to be rejected at the end of the study, e.g.,

$${P}_{\pi }\left({H}_{A},|,\text{Data}\right) \,>\, 0.975.$$

where $p$ is the sensitivity or specificity of the autonomous AI system and ${p}_{0}=75 \%$ for the sensitivity endpoint and ${p}_{0}=77.5 \%$ for the specificity endpoint under the null hypotheses.

We pre-specified conservative one-sided non-inferior hypothesis testing with overall one-sided 2.5% Type I error and >80% power to rule out pre-defined 77.5% specificity and 75% sensitivity lower bounds, using clustered bootstrapping as the primary analysis methodology to account for inter-eye correlation and randomly expanding the percent with ETDRS level ≥ 53 to be consistent with the target population. One-sided 97.5% lower confidence bounds were reported, except where indicated when 95% confidence intervals or standard deviation were used. Reported subgroup analyses were also prespecified; subgroups <10 participants are not reported. The primary and secondary endpoints were preregistered and prespecified on clinicaltrials.gov NCT05808699, and the detailed statistical analysis plan (SAP) was finalized before database lock. The SAP documents the sample size and power analysis for the primary endpoints – in a hypothesis testing design - analysis methods, data handling procedures, and other statistical analysis considerations. Bonferroni correction would be inappropriate for the primary endpoints. For secondary and exploratory endpoints, hierarchical testing was pre-specified in lieu of multiple testing correction, as others^65,66^ have noted the limitations of such adjustments. All calculations were performed using SAS statistical software, version 9.4.

## Supplementary information

Supplementary Information

## Data Availability

Data and materials availability: the Protocol, Statistical Analysis Plan, and STARD checklist, are available as Supplementary Information. The datasets generated during the current study that were used to calculate the primary outcome parameters are available upon reasonable request from the corresponding author, MDA, as well as from PTL. Code availability: the improved autonomous AI system described in this study is available as LumineticsGo from Digital Diagnostics, Coralville, Iowa. The underlying source codes are copyrighted by the sponsor, and are not available. No other custom code was used in the study.

## Code availability

The autonomous AI system described in this study is available as LumineticsGo from Digital Diagnostics, Coralville, Iowa. The underlying source codes are copyrighted by the sponsor, and are not available. No other custom code was used in the study.
