# Supplementary material for: Mitigation of AI adoption bias through an improved autonomous AI system for diabetic retinal disease
Source: NPJ Digit Med. 2024 Dec 19;7:369. doi: 10.1038/s41746-024-01389-x (PMC11659561; doi:10.1038/s41746-024-01389-x)
Supplement: Supplementary file 1 — Supplementary Information [file 41746_2024_1389_MOESM1_ESM.pdf]

CTMF-SA-DXSDR006-03	SAP_2.1_clean			
Issued by: Clinical Operations		Effective Date: 9/27/2023	Rev. A	Pg. 1 of 16

Approved: 9/25/2023 6:30 PM<br>Audrey Singh	Approved: 9/25/2023 9:51 AM<br>Abhay Shah	Approved: 9/26/2023 4:04 PM<br>Michael Abramoff	Approved: 9/25/2023 9:49 AM<br>Ashley Miller, Regulatory Affairs Manager

Qualification of a New Fundus Camera (RV700)
for An Autonomous AI System
for Detecting Diabetic Retinopathy and Diabetic Macular Edema

Version 2.1

September 19, 2023

Prepared by:

Philip Lavin, PhD, FASA, FRAPS

Cara J. Joyce, PhD MS

For:

Digital Diagnostics, Inc.

1. Introduction

Background: Diabetic retinopathy and Diabetic Macular Edema (DR) are the most common cause of blindness in the working population of the United States [1]. Each year, DR leads to more than 24,000 cases of preventable blindness in the United States. An estimated 4.1 million Americans are affected by DR including 899,000 with vision-threatening retinopathy [2].

Early detection of DR can prevent vision loss and blindness [3-7]; however, a large portion of individuals with diabetes do not currently undergo routine DR examinations [8]. In addition, specific disadvantaged groups have increased prevalence of diabetic retinopathy, where low rates of DR evaluation are at least partially associated with lack of access to a specialist exam [9].

FDA De Novo cleared IDx-DR, an autonomous artificial intelligence (AI) diagnostic system designed to detect DR in primary care [10]. Since its initial marketing in 2018, IDx-DR has been shown to address the unmet DR evaluation need, address the lack of access to care, and racial and ethnic disparities that have persisted for decades. IDx-DR was designed, developed, validated, and marketed under a strict ethical framework, which is documented in a series of publications through the Collaborative Community for Ophthalmic Imaging in which FDA participates [11]. IDx-DR is currently only indicated for use with one camera, Topcon NW400, for automatically detecting more than mild diabetic retinopathy (mtmDR), defined as ETDRS severity level 35 or higher, and or macular edema, in adult subjects diagnosed with diabetes not previously diagnosed with diabetic retinopathy.

FDA has requested a labeling extension to another camera as well as an eye-level analysis. This protocol and SAP addresses both needs.

Scope: The purpose of this study is to demonstrate that IDx-DR using the RetinaVue 700 Handheld Retinal Camera (RV700) is substantially equivalent to the predicate device, IDx-DR paired with the Topcon NW400, in terms of sensitivity and specificity, when compared at a per-eye level (a minimum of 200 eyes with mtmDR and 140 eyes without mtmDR). Success will be defined by demonstrating non-inferiority to the predicate device's performance levels at the eye-level. Additional analyses will be performed at the subject-level for regulatory submission and publication; this is consistent with the de novo study utilizing the Topcon NW400 retinal camera.

This study also includes a Precision Substudy to demonstrate the repeatability and reproducibility of IDx-DR with the RV700 using the same Latin Squares design as was used for the pivotal study. A total of 36 subjects who participated in the Diagnostic Performance Study will be invited to participate in the Precision Substudy. Of these 36 subjects, 18 will have received a PRS determination of mtmDR(+) for at least one eye, and 18 will have received a PRS determination of mtmDR(-) for both eyes. Each participating subject will undergo a series of nine imaging sessions with three different cameras, three operators, and three replicates to test the reproducibility and repeatability of using IDx-DR with the RV700. Analyses will be conducted at the eye-level as well as at the subject-level.

2. Study objectives and endpoints

Objectives	Endpoints
**Primary**	
1. To demonstrate the substantial equivalence of IDx-DR using the RV700 to the predicate device (IDx-DR using the NW400) in detecting ETDRS level 35 or higher and/or DME	1. Sensitivity and specificity of IDx-DR using RV700 based on eye-level results against a Level I Prognostic Reference Standard (PRS)<br>2. Eye-level sensitivity calculated using weighted sampling so that ETDRS level 53 or higher represents 20% of the mtmDR(+) sample
**Secondary**	
1. To determine IDx-DR (using RV700) diagnosability, the percentage of eyes for which a diagnostic result can be provided.<br>2. To determine IDx-DR (using RV700) diagnosability, the percentage of subjects for which a diagnostic result can be provided.<br>3. To determine the positive and negative predictive values.<br>4. To investigate the effect of cases where no diagnostic result can be provided on sensitivity and specificity using a "worst case scenario" analysis approach.	1. Sensitivity and specificity of IDx-DR using RV700 based on subject-level results against a Level I Prognostic Reference Standard (PRS)<br>2. Sensitivity and specificity of IDx-DR using RV700 based on eye-level results when compared to the Wisconsin Fundus Photograph Reading Center Level II Standard grading of the RV700 images<br>3. Sensitivity and specificity of IDx-DR using RV700 based on subject-level results when compared to the Wisconsin Fundus Photograph Reading Center Level II Standard grading of the RV700 images<br>4. Percent of total cases where a diagnostic result can be provided by IDx-DR with the RV700<br>5. Positive Predictive Value (PPV) and Negative Predictive Value (NPV) of IDx-DR with the RV700; repeat PPV and NPV calculations using the above weighted samples<br>6. Sensitivity and specificity of IDx-DR categorizing all PRS-determined positive cases with no diagnostic result as false negatives and all PRS-determined negative cases with no diagnostic result as false positives, i.e., a "worst case scenario" analysis of the impact of "Exam Quality Insufficient" results
**Precision Substudy**	

Company Confidential - Not for Public Disclosure

1. To evaluate the eye-level repeatability and reproducibility of IDx-DR with the RV700.<br>2. To evaluate the subject-level repeatability and reproducibility of IDx-DR with the RV700.	1. Eye-level DDx algorithm score mixed model estimates of the operator, camera, sequence, dilation, and FPRC effects<br>2. Average positive agreement (APA) in intra- and inter-configurations of RV700 cameras/operators<br>3. Average negative agreement (ANA) in intra- and inter-configurations of RV700 cameras/operators<br>4. Average ungradable agreement (AUA) in intra- and inter-configurations of RV700 cameras/operators<br>5. Overall agreement<br>6. Subject-level DDx algorithm mixed model estimates of the operator, camera, sequence, dilation, and FPRC effects

2.1 Diagnostic Performance Study Primary endpoints: The primary objective of this study is to estimate the sensitivity and specificity of IDx-DR with the RV700 in detecting mtmDR based on eye-level results when compared to a prognostic reference standard. For each eye from each subject enrolled, IDx-DR results will be obtained as positive for mtmDR (hereafter, positive), negative for mtmDR (negative), or exam quality insufficient/failed (ungradable). Similarly, results of positive, negative, or ungradable based on Level I grading will be determined by the majority vote of retinal experts at a reading center as the prognostic reference standard (ETDRS level 35 or higher and/or DME). The unit of analysis will be the eye, and confidence intervals that account for inter-eye correlation will be presented as a measure of precision for all point estimates. The non-inferiority targets will be set based on Sponsor expectations for eye-level sensitivity and specificity. Published results of this trial are in **Supplementary Table 1**.

Supplementary Table 1: Eye-level diagnostic design parameters and pivotal study subject-level performance

	Eye-level Null Hypothesis p_0	Subject-level Point Estimate (two-sided 95% CI)
Sensitivity	75.0%	87.2% (81.8-91.2%)
Specificity	77.5%	90.7% (88.3-92.7%)

The primary efficacy analysis for the eye-level sensitivity analysis will be repeated for Level 1 using overweight sampling to achieve 20% mtmDR(+) with ETDRS level 53 or higher.

In addition, the primary analyses will be performed at the subject-level for regulatory submission and publication; this is consistent with the de novo study utilizing the Topcon NW400 retinal camera.

Company Confidential - Not for Public Disclosure

2.2 Diagnostic Performance Study Secondary endpoints: Secondary endpoints include estimation of test characteristics including positive predictive value (PPV), negative predictive value (NPV), and diagnosability. Other secondary endpoints include calculations of sensitivity and specificity that include each test's respective ungradable eyes in the denominator (see Supplementary Table 2 below). One-sided 97.5% confidence intervals that account for inter-eye correlation will be presented as a measure of precision for all point estimates.

For secondary efficacy endpoints, the following will be analyzed:

1) Diagnosability: the percentage of total cases where a diagnostic result can be provided by IDx-DR with the RV700
2) Positive Predictive Value (PPV) and Negative Predictive Value (NPV) of IDx-DR with the RV700;
3) Sensitivity and specificity of IDx-DR as if all PRS-determined positive cases with no diagnostic result provided were false negatives and all PRS-determined negative cases with no diagnostic results were false positives, i.e., a "worst case scenario" analysis of the impact of "Exam Quality Insufficient" results
4) Sensitivity and specificity of IDx-DR using RV700 based on eye-level results when compared to the Wisconsin Fundus Photograph Reading Center (FPRC) Level II Standard grading of the RV700 images.

The corresponding PPV and NPV from weighted sampling will also be computed; the same specificity population will be used for these additional calculations.

2.3 Definitions for Primary and Secondary Endpoint Calculations

Supplementary Table 2

	Prognostic reference standard			
	Positive	Negative	Ungradable	Total
IDx-DR Result				
Positive	PP	PN	PU	DP
Negative	NP	NN	NU	DN
Ungradable	UP	UN	UU	DU
Total	TP	TN	TU	N

Endpoints:

$$\text{Sensitivity} = \frac{PP}{PP+NP} \qquad \text{Specificity} = \frac{NN}{PN+NN}$$

$$\text{Positive Predictive Value} = \frac{PP}{PP+PN} \qquad \text{Negative Predictive Value} = \frac{NN}{NP+NN}$$

$$\text{Diagnosibility} = \frac{PP+PN+NP+NN}{TP+TN}$$

Endpoints considering ungradable eyes:

$$\text{Sensitivity}_{+\text{u}} = \frac{PP}{PP+NP+UP} \qquad \text{Specificity}_{+\text{u}} = \frac{NN}{PN+NN+UN}$$

$$\text{PPV}_{+\text{u}} = \frac{PP}{PP+PN+PU} \qquad \text{NPV}_{+\text{u}} = \frac{NN}{NP+NN+NU}$$

2.4 Precision Substudy Primary Objective:

The primary objective of the precision substudy is to evaluate the repeatability and reproducibility of IDx-DR with the RV700.

Precision Substudy Endpoints:

For the Precision Substudy, the following will be analyzed:

1. Eye-level DDx algorithm mixed model estimates of the operator, camera, sequence, dilation, and FPRC effects
2. Average positive agreement (APA) in intra- and inter- configurations of RV700 cameras/operators
3. Average negative agreement (ANA) in intra- and inter- configurations of RV700 cameras/operators
4. Average ungradable agreement (AUA) in intra- and inter- configurations of RV700 cameras/operators
5. Overall agreement
6. Subject-level DDx algorithm mixed model estimates of the operator, camera, sequence, dilation, and FPRC effects.

3. Design and methods

3.1 Cohort: This is a Diagnostic Performance Study, where a minimum of 200 eyes with mtmDR and at least 140 eyes without mtmDR will be enrolled at primary care sites. The study population is expected to mirror the spectrum of disease severity encountered in primary care settings, from no DR to proliferative DR (PDR), with a target sample size of at least 200 eyes (~20% of subject sample size) with more than mild DR (mtmDR+), which includes ETDRS levels 37-85, and/or clinically significant as well as center-involved macular edema as subgroup analyses. The study population is also expected to mirror the racial and ethnic distribution of the US diabetes population. Within this cohort, at least 20 eyes are expected to have vision

threatening DR (vtDR), defined as ETDRS level 53-85 and/or clinically significant as well as center-involved macular edema; overweighted sampling will be used to meet this vtDR goal.

The overweight sampling will be performed with a sample of 1,000 random draws to expand the number of ETDRS eyes with the level of at least 53 from a minimum of 20 eyes to represent 20% mtmDR(+). The minimum of 20 eyes must be reading center gradable and sufficient, Specificity calculations will rely on those eyes with ETDRS level less than 37. The sampling will be conducted before any analyses are performed.

3.2 Precision Substudy sample: Select subjects will be invited for the Precision Substudy based on the PRS grading of their Diagnostic Performance Study images. Eighteen (18) of the Precision substudy subjects will have received a grade of mtmDR(+) in at least one eye, and eighteen (18) will have received a grade of mtmDR(-) in both eyes. Subjects will be fully dilated and then undergo nine IDx-DR exams. The substudy will test three RV700 cameras, three operators, and three replicates using a Latin Square design. The camera, operator, and replicate combinations across subjects are specified below:

Exam A: RV700 A + Operator #1 (Replicate 1)
Exam B: RV700 A + Operator #2 (Replicate 1)
Exam C: RV700 A + Operator #2 (Replicate 2)
Exam D: RV700 B + Operator #2 (Replicate 1)
Exam E: RV700 B + Operator #3 (Replicate 1)
Exam F: RV700 B + Operator #3 (Replicate 2)
Exam G: RV700 C + Operator #1 (Replicate 1)
Exam H: RV700 C + Operator #1 (Replicate 2)
Exam I: RV700 C + Operator #3 (Replicate 1)

The Latin Squares design follows where the X represents an observation and R represents a replicate in order to estimate camera effect, operator effect, and reproducibility:

	Operator 1	Operator 2	Operator 3
Camera A	X	X, R	-
Camera B	-	X	X, R
Camera C	X, R	-	X

Operators will reposition subjects between each imaging replicate. The order of the data collection will be recorded in the case report form.

4. Sample size calculations

Assumptions: In this study, a minimum of 200 eyes with mtmDR and at least 140 eyes without mtmDR will be enrolled in the main diagnostic performance study; enrollment will be continued until a minimum of 20 vtDR eyes are confirmed to be sufficient and gradable. The device performance will be evaluated per eye. The primary endpoints will be the sensitivity and specificity of IDx-DR paired with the RV700 in detecting mtmDR based on eye-level results when compared to a PRS by three retinal experts.

The predicate device, IDx-DR with the NW400, pivotal trial pre-specified null hypotheses for sensitivity and specificity of 75% and 77.5%, respectively; these represent pre-defined non-inferiority margins. The alternative hypotheses were 85.0% for sensitivity and 82.5% for specificity in support of a non-inferiority design. These lower thresholds for performance will be ruled out with one-sided 97.5% confidence bounds.

As this study is intended to support an extension of the IDx-DR labeling to validate a new camera for use with IDx-DR, the performance is expected to be substantially equivalent to the predicate device at the subject-level.

This study will validate the use of a new camera with the same diagnostic algorithm as currently on the market. The proposed sample size maintains a one-sided 97.5% confidence interval that exceeds the predicate device pivotal trial null hypotheses, i.e., a lower confidence limit > 75% for sensitivity and > 77.5% for specificity which reflects a 5% non-inferiority offset for sensitivity and a 2.5% non-inferiority offset for specificity (see **Supplementary Table 3** columns 1-4). These hypothesis tests were designed specifically to reflect clinically meaningful eye-level outcomes; substantial equivalence is interpreted as non-inferiority
(**Supplementary Table 3** columns 1-4).

Supplementary Table 3: Power and Significance Threshold Calculations Summary (minimum sample sizes)

	Sponsor Criteria (Non-inferiority)			
	Sensitivity		Specificity	
	Power	N-I	Power	N-I
	**1**	2	3	4
Test significance level, α	0.025	0.025	0.025	0.025
1 or 2 sided test?	1	1	1	1
Null hypothesis %, π_0	75%	75%	77.5%	77.5%
Alternative hypothesis %, π_A	83.5%	81.2%	87.1%	84.6%
Power (%)	80	NA	80	NA
N	200	200	140	140

Supplementary Table 4 summarizes the study design parameters inclusive of:

- the eye-level hypotheses to be tested (non-inferiority)
- the non-inferiority thresholds and offsets
- the sample size requirements, and
- the thresholds needed to establish non-inferiority.

Supplementary Table 4: Inputs to power calculations for one-sided exact binomial tests

Sensitivity	***Specificity***
H0: P ≤ 0.75 vs. H1: P > 0.75	H0: P ≤ 0.775 vs. H1: P > 0.775
$p_{baseline} = 0.80$ is the sensitivity target	$p_{baseline} = 0.80$ is the specificity target
$p_0 = 0.75$ sensitivity values below 75% are considered inferior to IDx-DR with NW400	$p_0 = 0.775$ specificity values below 77.5% are considered inferior to IDx-DR with NW400
$p_1 = 0.835$ hypothesized sensitivity of IDx-DR with RV700	$p_1 = 0.871$ hypothesized specificity of IDx-DR with RV700

$d_0 = -0.05$ non-inferiority difference.<br>$d_0 = p_0 - p_{baseline}$ and is the maximum distance that may still be considered non-inferior.	$d_0 = -0.025$ non-inferiority difference.<br>$d_0 = p_0 - p_{baseline}$ and is the maximum distance that may still be considered non-inferior.
$d_1 = 0.085$ is the difference at which power is calculated. $d_1 = p_1 - p_0$ and is the hypothesized sensitivity value above the non-inferiority threshold	$d_1 = 0.096$ is the difference at which power is calculated. $d_1 = p_1 - p_0$ and is the hypothesized specificity value above the non-inferiority threshold
n=200 mtmDR(+) eyes	n=140 mtmDR(-) eyes
Reject H_0 for at least 163/200 true positive eyes, or at least 81.2% sensitivity.<br>Establish superiority for at least 172/200 true positive eyes, or at least 85.7% sensitivity.	Reject H_0 for at least 119/140 true negative eyes, or at least 84.6% specificity.<br>Establish superiority for at least 122/140 true-negative eyes, or at least 86.7% specificity.

The mtmDR(+) and mtmDR(-) sample size depends on the WRC findings so the study will enroll to a target of 564 eyes (282 subjects maximum) to ensure sufficient numbers for the sensitivity and specificity analyses. In addition, in order to achieve 200 mtmDR(+) eyes, we anticipate >450 mtmDR(-) eyes will be accrued based on the historical enrollment ratio (1:4). Thus, the specificity power will be increased and the threshold to establish non-inferiority and superiority will be reduced. Similarly, in the event that >200 mtmDR(+) eyes are determined, all eyes will be included. Thus, the plan is to include all recruited subjects in the final analyses in support of the intent to diagnose analysis principle.

Additionally, weighted sampling will be performed by sampling with replacement among eyes with ETDRS levels at least 53 for computing sensitivity, PPV, and NPV in settings with higher prevalence of severe disease. The overweight sampling is expected to achieve similar sensitivity due to the samples being drawn at random from the gradable and sufficient eyes with ETDRS greater than 53. This analysis is being done following discussion with FDA to assess PPV and NPV outcomes in populations with different vision threatening incidence.

Simulation studies were performed using the clustered bootstrap approach for confidence interval calculations [12] and similar power levels were achieved to the exact binomial method.

The study will not be powered to test specific subject-level hypotheses; the retrospective power will be computed consistent with the pivotal study performance.

5. Analysis

5.1 Diagnostic Performance Study: A cross tabulation of IDx-DR results (positive, negative, ungradable) with the results from the reference standard (positive, negative, ungradable) will be presented for the overall sample. The point estimate for each test characteristic will be presented along with the counts of eyes (numerator and denominator) that contribute to the calculation. One-sided 97.5% confidence intervals will be computed by the cluster bootstrap approach to account for correlation introduced by taking measurements from both eyes of each subject; in cases where the confidence interval is not estimable using the cluster bootstrap approach due to small sample sizes or boundary values (e.g., a test characteristic of 100%), the exact binomial confidence interval will be presented instead. Primary endpoints will be reported in stratified

analyses by eye, age, sex, and race/ethnicity. Should enrichment be implemented, further stratification of all endpoints will be presented by cohort: sequential enrollment or enrichment.

The sensitivity, PPV, and NPV analyses will be repeated in the same manner for the weighted samples. The PPV and NPV calculations will be performed using the accrued specificity data without any resampling of eyes with ETDRS level less than 53.

Relevant data analyses will be repeated at the subject-level for regulatory submission and publication consistent with the de novo study utilizing the Topcon NW400 retinal camera.

Data analysis will be performed using SAS (V9.3 or later).

Population: We anticipate those recruited will mirror the spectrum of disease severity encountered at primary care settings and be similar to the study population in the pivotal trial. [10] In the analyzable population, 30.9% were ≥ 65 years old, 16.4% were Hispanic, 28.2% Black, and 86.7% had HbA1c ≥ 7.

Protocol deviations: The contract research organization (Labcorp) will track the progress of subject enrollment and data management, calculating totals per stratum on a monthly basis. While all efforts will be made to minimize the likelihood of protocol deviations through site training on all procedures, potential deviations will be monitored throughout the study. A non-exhaustive list of potential deviations includes:

- failure to obtain signed informed consent before initiating study-related procedures
- an operator taking more than 6 attempts per eye
- an operator failing to take at least 6 attempts without a diagnostic result.

Some protocol deviations may result in participant data excluded from endpoint analysis, while others may not require exclusion of the participant data. Hypothesized deviations and intended actions are described in **Supplementary Table 5**, and any new deviations will similarly be outlined with an action plan prior to formal statistical analysis.

Supplementary Table 5: Protocol deviations

Deviation	Action
Failure to obtain signed informed consent before initiating study-related procedures	Exclude participant data from endpoint analysis
More than 6 attempts per eye	Only data from the first 6 attempts may be included in endpoint analysis (exclude attempt 7 or greater)
Failing to make at least 6 attempts without a diagnostic result	Exclude participant data from endpoint analysis
Failure to capture both eyes for a participant	The single eye may be included should no other protocol deviations be associated with the participant's data collection.

After enrollment, an eligibility criterion not met per protocol	Exclude participant data from endpoint analysis

In all cases of protocol deviations, the site will be notified of the deviation to prevent future such occurrences. The final analytic sample size for primary endpoints will be described with counts and reasons for exclusion (protocol deviations, reading center ungradable, or IDx-DR ungradable) in a flow diagram as in **Figure 1**.

Figure 1: Example flow diagram

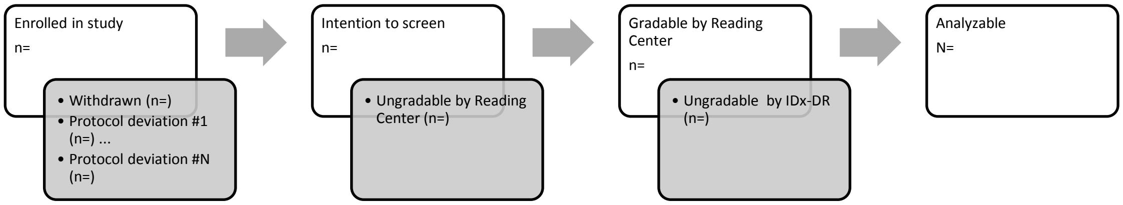

***Baseline Covariates*:** Data collection for each eye will include a unique participant identifier plus age in years at exam, sex, race, ethnicity, ocular condition/procedure history, non-ocular comorbid conditions, diabetic status including duration of diagnosis, recent lab tests (HbA1c, fasting glucose). Continuous variables will be summarized using descriptive statistics (means, standard deviations, and quartiles), and nominal variables will be summarized using counts and percentages. Primary endpoints may be reported in stratified analyses by age, sex, and race/ethnicity. Demographic variables will be presented at the subject-level and eye-level according to: (1) not mtmDR or vtDR, (2) mtmDR, and (3) vtDR.

For the weighted sampling, the demographics (means, SDs) will also be repeated to include those subjects with eyes selected from overweight sampling.

Missing Data: By design, each eye will be represented in a cell in the crosstabulation depicted in Section 2.3; missing data are not anticipated. Any missing or ungradable data will be displayed; all available eye-level data will be used. The primary endpoints will be assessed where both the PRS and IDx-DR results will be gradable. Secondary "worst case scenario" analysis considers ungradable values within the calculation.

Statistical Analysis: A cross-tabulation of IDx-DR results (positive, negative, ungradable) with the results from the reference standard (positive, negative, ungradable) will be presented for the overall sample. The point estimate for each test characteristic will be presented along with the counts of eyes (numerator and denominator) that contribute to the calculation. Should enrichment be implemented, further stratification will report point estimates and counts of eyes (numerator and denominator) by cohort (not enriched, enriched).

Eye-level one-sided 97.5% lower confidence intervals will be computed by the cluster bootstrap approach to account for correlation introduced by taking measurements from both eyes of each subject [12]; specifically:

- participants will be stratified by the number of eyes contributing to the analysis (1 or 2) and by the number of eyes with mtmDR(+) (0, 1, or 2)
- sampling with replacement will be performed within each stratum until the same number of participants per stratum are selected
- all eligible eyes per participant selected in the bootstrap sample will be included to estimate the point estimate in the bootstrapped sample
- this process will be repeated 1000 times, with the empirical 95% confidence interval reported as the 25th and 975th ordered point estimate from bootstrapped samples
- in case the confidence interval is not estimable using the cluster bootstrap approach due to small sample sizes or boundary values (e.g., a test characteristic of 100%), the exact binomial confidence interval will be presented instead.
- Weighted sampling will also be conducted to assess the impact of prevalence upon sensitivity, PPV, and NPV. This is important to ensure that these outcomes can be extrapolated to populations with higher proportions of eyes with ETDRS level at least 53.

In addition, eye-level logistic regression models will be performed to account for correlations and the same baseline covariates. The model-based estimates will be corrected to address the estimate bias introduced by using logs introduced by logistic regression.

Relevant data analyses will be repeated at the subject-level for regulatory submission and publication consistent with the de novo study utilizing the Topcon NW400 retinal camera.

Reporting: The CONSORT-AI [Consolidated Standards of Reporting Trials–Artificial Intelligence] extension to the 2010 CONSORT statement provides guidelines for reporting interventions involving artificial intelligence [13]. The 25 item CONSORT 2010 checklist includes 14 new items with additional detail about the AI component. The checklist will be completed by the study statistician with input from the study sponsor and will be included in any publication resulting from this trial.

5.2 Precision Substudy: The repeatability and reproducibility substudy will be performed including three cameras and three operators (see Section 3.2). A Latin Squares design (Supplementary Table **6**) will be used with mixed effects models used to analyze the DDx algorithm outcome data on a log-scale with operator, camera, sequence, dilation, and FPRC as covariates. Separate analyses will be performed at the subject-level and at the eye-level. Each of 36 subjects, 50% positive for mtmDR in at least one eye, will have a total of 9 results on each eye (see protocol Section 9.4.1). Repeatability will be assessed within the three replicates from pre-specified camera-operator pairs, and reproducibility will be assessed from comparing results across the six camera-operator combinations. There will be 3 replicates on all 36 subjects (72 eyes) for 216 gradings for the replicate analyses and 6 measures on all 36 subjects (72 eyes) for 432 gradings for the reproducibility analysis.

Supplementary Table 6: Imaging Combinations for Precision Substudy

Exam	Camera	Operator	Replicate
A	A	1	1
B		2	1
C		2	2
D	B	2	1
E		3	1
F		3	2
G	C	1	1
H		1	2
I		3	1
	Subjects per setting		9

Endpoints for each analysis include overall agreement, average positive agreement, average negative agreement, and average ungradable agreement. DDx algorithm repeatability, or intra-operator variability, will be assessed using mixed effects models to simultaneously estimate these three separate sources of variation (see protocol Section 9.4.2). One-sided 97.5% confidence intervals for each endpoint will be computed using the clustered bootstrap approach. [12]

Relevant data analyses will be repeated at the subject-level for regulatory submission and publication consistent with the de novo study utilizing the Topcon NW400 retinal camera.

Cross-Tabulation for Agreement Statistics

	R2 Positive	R2 Negative	R2 Ungradable
R1 Positive	PP	PN	PU
R1 Negative	NP	NN	NU
R1 Ungradable	UP	UN	UU

R1 and R2 represent replicates 1 and 2

Formulas:

$$\text{Overall agreement (OA)} = \frac{PP+NN+UU}{Total}$$

$$\text{Average positive agreement (APA)} = \frac{2PP}{(PP+NP+UP)+(PP+PN+PU)}$$

$$\text{Average negative agreement (ANA)} = \frac{2NN}{(PN+NN+UN)+(NP+NN+NU)}$$

$$\text{Average ungradable agreement (AUA)} = \frac{2UU}{(PU+NU+UU)+(UP+UN+UU)}$$

6. References

1. Klonoff, D.C. and D.M. Schwartz, An economic analysis of interventions for diabetes. Diabetes Care, 2000. 23(3): p. 390-404.
2. CDC. Vision Health Initiative. 2015 [cited 2015 August 4, 2015]; Available from: http://www.cdc.gov/visionhealth/basic_information/eye_disorders.htm#a5.
3. Bressler, N.M., et al., Underuse of the health care system by persons with diabetes mellitus and diabetic macular edema in the United States. JAMA Ophthalmol, 2014. 132(2): p. 168-73.
4. Early photocoagulation for diabetic retinopathy. ETDRS report number 9. Early Treatment Diabetic Retinopathy Study Research Group. Ophthalmology, 1991. 98(5 Suppl): p. 766-785.
5. Photocoagulation for diabetic macular edema. Early Treatment Diabetic Retinopathy Study report number 1. Early Treatment Diabetic Retinopathy Study research group. Arch.Ophthalmol., 1985. 103(12): p. 1796-1806.
6. Diabetic Retinopathy Study, G., Photocoagulation treatment of proliferative diabetic retinopathy: clinical application of DRS findings: DRS report 8. Ophthalmology, 1981. 88: p. 583-600.
7. Diabetic Retinopathy Study, G., Indications for photocoagulation treatment of diabetic retinopathy: DRS report 14. Int Ophthalmol Clin, 1987. 27: p. 239-253.
8. Benoit, S.R., et al., Eye Care Utilization Among Insured People With Diabetes in the U.S., 2010–2014. Diabetes Care, 2019 March. 42(3): p. 427-433.
9. Lee, D.J., et al., Dilated eye examination screening guideline compliance among patients with diabetes without a diabetic retinopathy diagnosis: the role of geographic access. BMJ Open Diabetes Res Care, 2014. 2(1): p. e000031.
10. Abràmoff, M.D., Lavin, P.T., Birch, M. et al. Pivotal trial of an autonomous AI-based diagnostic system for detection of diabetic retinopathy in primary care offices. npj Digital Med 1, 39 (2018).
11. Abramoff, M.D., Cunningham, B, Patel, P, Eydelman, MB, Leng, T, et al. Foundational Considerations for Artificial Intelligence Using Ophthalmic Images. Ophthalmology 2022 Feb;129(2):e14-e32.

12. Ying GS, Maguire MG, Glynn RJ, Rosner B. Calculating sensitivity, specificity, and predictive values for correlated eye data. Investigative ophthalmology & visual science. 2020 Sep 1;61(11):29.
13. Campbell JP, Lee , AY, Abràmoff MD, Pearse, KA , Ting DS, et al. Reporting Guidelines for Artificial Intelligence in Medical Research. Ophthalmology. 2020 Dec;127(12):1596-1599.
14. Liu X, Rivera SC, Moher D, Calvert MJ, Denniston AK. Reporting guidelines for clinical trial reports for interventions involving artificial intelligence: the CONSORT-AI Extension. bmj. 2020 Sep 9;370.

Revision History

Revision Number	Revision Summary
1	Initial release
2.0	• Clarification that the analyses will be conducted at eye-level and subject-level.<br>• Addition of secondary objective pertaining to IDx-DR (using RV700) diagnosability, the percentage of eyes for which a diagnosability result can be provided.<br>• Addition of two secondary endpoints pertaining to sensitivity and specificity of IDx-DR using RV700 based on subject level results at level 1 and level II.<br>• Updated the precision substudy to state the use of Latin square design.<br>• Updated sample size calculations (Section 4 of the SAP) and added clarifying language to this section.
2.1	• Clarification on number of eyes expected to have vtDR, and the minimum vtDR eyes required to be sufficient and gradable.<br>• Inclusion of overweighted sampling to meet vtDR goal.

Phil Lavin

Cara Joyce

Phil Lavin

Cara Joyce

September 21, 2023

September 21, 2023

**PROTOCOL NUMBER:**	**DXSDR006**
**TITLE:**	Qualification of a New Fundus Camera Paired with An Autonomous AI for Detecting Diabetic Retinopathy
**STUDY PHASE:**	Pre-Market
**INVESTIGATIONAL PRODUCT:**	IDx-DR paired with RV700 fundus camera
**IDE #**	NA
**CLINICALTRIALS.GOV#**	Pending
**SPONSOR:**	Digital Diagnostics, Inc.<br>210 5th Street, Suite 103 .<br>Coralville, IA 52241<br>319-248-5620
**STUDY DIRECTOR:**	Audrey Singh, Director of Clinical Operations<br>Digital Diagnostics Inc.
**STUDY MEDICAL DIRECTOR:**	Michael Abramoff, MD, PhD<br>Digital Diagnostics Inc.
**PROTOCOL VERSION:**	Version 3.0
**VERSION DATE:**	20-March-2023

CONFIDENTIAL NOT FOR DISTRIBUTION

(see confidentiality statement)

Digital Diagnostics Confidential. This document is intended for internal use only and may not be disclosed or reproduced in whole, or in part, for external distribution in any form without express written permission of DDx Legal. All hard copies should be checked against the current electronic revision prior to use and destroyed promptly thereafter. All hard copies are considered uncontrolled documents.

SPONSOR SIGNATORY

This Version 3.0 of protocol number **DXSDR006** has been prepared and reviewed by the Sponsor for distribution to designated clinical sites, associated Institutional Review Boards (IRBs)/Ethics Committees, designated contractors, regulatory agencies, with permission by the Sponsor. This version of the protocol shall supersede all previous versions of the specified protocol.

____ ___________________________

Audrey Singh Date

Director of Clinical Operations, Digital Diagnostics

Medical Monitor Name and Contact Information

Michael Abramoff, MD, PhD
Digital Diagnostics Inc.
(319) 248-5620
abramoff@digitialdiagnostics.com

Digital Diagnostics Confidential. This document is intended for internal use only and may not be disclosed or reproduced in whole, or in part, for external distribution in any form without express written permission of DDx Legal. All hard copies should be checked against the current electronic revision prior to use and destroyed promptly thereafter. All hard copies are considered uncontrolled documents.

CONFIDENTIALITY STATEMENT

This document contains confidential information that is proprietary to Digital Diagnostics Inc. (DDx). You may only disclose the contents to study personnel under your supervision who need to know the contents for the purpose of conducting a clinical study, in addition to your IRB or other Committees for evaluation of the clinical study as appropriate under the following condition: the contents of this document must remain confidential and may not be disclosed to any other person or entity without prior written permission from DDx. This will not apply to disclosure that is required by governmental regulations or laws; however, you must promptly notify Digital Diagnostics of any such disclosure. Any supplemental information that may be added or appended to this document is also confidential and proprietary to Digital Diagnostics and must be treated in the same manner.

Digital Diagnostics Confidential. This document is intended for internal use only and may not be disclosed or reproduced in whole, or in part, for external distribution in any form without express written permission of DDx Legal. All hard copies should be checked against the current electronic revision prior to use and destroyed promptly thereafter. All hard copies are considered uncontrolled documents.

INVESTIGATOR ACKNOWLEDGEMENT

**Study Title:** Qualification of a New Fundus Camera Paired with An Autonomous AI for Detecting Diabetic Retinopathy<br><br>**Protocol Number and Version:** DXSDR006, Version 3.0

I have received and read this protocol **DXSDR006**, including all appendices. I agree to abide by all the provisions set forth in this protocol. I agree to conduct the study in compliance with any applicable US FDA regulations (e.g., 21 CFR Parts 50, 54, and 56), Good Clinical Practice (GCP) as defined in the ICH guidelines and any locally applicable regulations. I agree to maintain the confidentiality of all information received or developed in connection with this protocol.

I agree that, prior to the commencement of this study, I must obtain all required Institutional approvals, including Independent Ethics Committee/ Institutional Review Board associated with the clinical facilities where the study will be conducted for this protocol and the informed consent document.

I will provide all study personnel under my supervision with copies of this protocol and access to all study-related information provided by the Sponsor. I will discuss this study-related information with my staff to ensure that they are fully informed about the investigational product and the protocol. I will be responsible for overall study conduct and agree to personally conduct or supervise all study activities at the clinical facilities where the study will be conducted.

I agree to provide all Subjects with a signed and dated copy of the informed consent document, as required by FDA regulations and ICH GCP. I further agree to report to Digital Diagnostics any adverse events in accordance with the terms of this protocol.

______________________________ ________________

Principal Investigator Printed Name Site Number

________________________________ ____________________

Principal Investigator Signature Date

Digital Diagnostics Confidential. This document is intended for internal use only and may not be disclosed or reproduced in whole, or in part, for external distribution in any form without express written permission of DDx Legal. All hard copies should be checked against the current electronic revision prior to use and destroyed promptly thereafter. All hard copies are considered uncontrolled documents.

TABLE OF CONTENTS

1. PROTOCOL SUMMARY 9
1.1. Synopsis 9
1.2. Diagnostic Performance Study Workflow 14
1.2.1. Diagnostic Performance Study Overall Schema 15
1.2.2 Diagnostic Performance Study's RV700 Imaging & AI Procedure 16
1.2.3 Diagnostic Performance Study Schedule of Activities 17
1.2.4 Diagnostic Performance Study Subject Identification 17
1.3. Precision Substudy Workflow 17
1.3.1. Precision Substudy Overall Schema 18
1.3.2. Precision Substudy Imaging Sequences .... Error! Bookmark not defined.
1.3.3. Precision Substudy IDx-DR Exam Procedure 19
1.3.4 Precision Substudy Camera Operator Configurations 20
1.3.5 Precision Substudy Schedule of Activities 20
1.3.6 Precision Substudy Subject and IDx-DR Exam Identification 20
2. INTRODUCTION 21
2.1. Background 21
2.2. Study Rationale 22
2.3. Benefit/Risk Assessment 22
3. OBJECTIVES AND ENDPOINTS 23
3.1. Diagnostic Performance Study 23
4. STUDY DESIGN 24
4.1. Overall Design 24
4.2. Scientific Rationale for Study Design 25
4.3. IDx-DR: Autonomous AI Device 26
5. STUDY POPULATION 27
5.1. Clinical Site Selection 27
5.1.1 Clinical Site Selection 27
5.1.2 Investigator Training 28
5.2. Screening 28
5.3. Inclusion Criteria 29
5.4. Exclusion Criteria 29
5.5. Enrollment 30
5.6. Screen Failures 30
5.7. Enrichment Inclusion and Exclusion Criteria 30
5.7.1 Inclusion criteria for enrichment: 30
5.7.2 Exclusion criteria for enrichment: 31
5.8. Subject Discontinuation/Withdrawal from the Study 31

Digital Diagnostics Confidential. This document is intended for internal use only and may not be disclosed or reproduced in whole, or in part, for external distribution in any form without express written permission of DDx Legal. All hard copies should be checked against the current electronic revision prior to use and destroyed promptly thereafter. All hard copies are considered uncontrolled documents.

5.9. Off Study/End of Study Definition 31
5.9.1. Off Study 31
5.9.2. End of Study 31
5.10. Discontinuation of Entire Study 32
6. DEVICES USED IN THIS STUDY 32
6.1. Fundus Camera to be Used in this Study 32
6.2. IDx-DR 32
6.2.1. IDx-DR Client 32
6.2.2. IDx-Service 33
6.2.3. IDx-DR Analysis 33
6.3. Preparation/Handling/Storage of Devices 33
6.4. Measures to Minimize Bias 33
7. STUDY ASSESSMENTS AND PROCEDURES 34
7.1. Screening 34
7.2. Diagnostic and Demographic Data 34
7.2.1. Collection of Race and Ethnicity Data 35
7.3. Representation Across Disease Spectrum 35
7.4. Enrichment Cohort 36
7.5. PRS Diagnostic Performance Study RV700 Imaging & AI Procedure 36
7.6 Precision Substudy IDx-DR Exam Procedure 37
8. CRITERIA FOR EVALUATION 38
8.1. Primary Efficacy Endpoint 38
8.2. Safety Assessments 39
8.2.1. Safety Monitoring 39
8.2.2. Methods and Timing for Assessing, Recording, and Analyzing Safety Parameters 40
8.2.3. Identification of Events and Timeframe for Reporting 41
8.2.4. Follow-up of Adverse Device Effects 41
8.2.5. Guidelines for Assessing Intensity of an Adverse Device Effect 41
8.2.6. Guidelines for Determining Causality 42
8.2.7. Reporting Procedures 42
8.2.8. Anticipated Adverse Device Effects 42
8.2.9. Unanticipated Adverse Device Effects 42
8.2.10. Protocol Deviations 42
8.2.11. Regulatory Reporting 43
8.2.12. Type and Duration of Follow-up of Subjects after ADEs 43
9. STATISTICAL CONSIDERATIONS 43
9.1. Hypothesis testing 43
9.2. Demographics 45
9.3. Endpoints 45
9.3.1. Primary Endpoint 45

Digital Diagnostics Confidential. This document is intended for internal use only and may not be disclosed or reproduced in whole, or in part, for external distribution in any form without express written permission of DDx Legal. All hard copies should be checked against the current electronic revision prior to use and destroyed promptly thereafter. All hard copies are considered uncontrolled documents.

9.3.2. Secondary Endpoints 45
9.3.3. Definitions for Primary and Secondary Endpoint Calculations 46
9.3.4. Analyses 47
9.4. Precision Substudy 47
9.4.1. Imaging Sequence for Precision Study 48
9.4.2. 3 by 3 by 3 Cross-Tabulation for Agreement Statistics 49
10. REGULATORY, ETHICAL, AND STUDY OVERSIGHT CONSIDERATIONS 50
11. REFERENCES 55
12. REVISION HISTORY 55

Digital Diagnostics Confidential. This document is intended for internal use only and may not be disclosed or reproduced in whole, or in part, for external distribution in any form without express written permission of DDx Legal. All hard copies should be checked against the current electronic revision prior to use and destroyed promptly thereafter. All hard copies are considered uncontrolled documents.

LIST OF ABBREVIATIONS

**AAO**	American Academy of Ophthalmology
**ADA**	American Diabetes Association
**ADE**	Adverse Device Effect
**AE**	Adverse event
**AI**	Artificial intelligence
**CFR**	Code of Federal Regulations
**CRO**	Contract Research Organization
**CSME**	Clinically Significant Diabetic Macular Edema
**DDx**	Digital Diagnostics, Inc.
**DME**	Diabetic Macular Edema
**DR**	Diabetic retinopathy
**DTP**	Data Transfer Plan
**eCRF**	Electronic Case Report Form
**EDC**	Electronic Data Capture
**ETDRS**	Early Treatment of Diabetic Retinopathy Study
**FDA**	Food and Drug Administration
**FPG**	Fasting Plasma Glucose
**FPRC**	Fundus Photograph Reading Center
**GCP**	Good Clinical Practice
**HbA1c**	Hemoglobin A1c
**HIPAA**	Health Insurance Portability and Accountability Act
**ICDR**	International Clinical Diabetic Retinopathy
**ICF**	Informed Consent Form
**ICH**	International Council on Harmonization
**IDE**	Investigational Device Exemption
**IRB**	Institutional Review Board
**ITS**	Intent to screen
**M**	Mean
**mtmDR**	More than mild diabetic retinopathy
**OCT**	Optical Coherence Tomography
**OGTT**	Oral glucose tolerance test
**OPC**	Objective performance criterion
**PDT**	Photodynamic therapy
**PG**	Plasma glucose
**PPP**	Preferred practice pattern
**PRS**	Prognostic Reference Standard
**RPG**	Random plasma glucose
**SAE**	Serious adverse event
**SAP**	Statistical Analysis Plan
**SD**	Standard deviation
**UADE**	Unanticipated adverse device effect
**WHO**	World Health Organization

Digital Diagnostics Confidential. This document is intended for internal use only and may not be disclosed or reproduced in whole, or in part, for external distribution in any form without express written permission of DDx Legal. All hard copies should be checked against the current electronic revision prior to use and destroyed promptly thereafter. All hard copies are considered uncontrolled documents.

**WRC**	Wisconsin Reading Center

1. Protocol Summary

1.1. Synopsis

**Protocol Title**	Qualification of a New Fundus Camera Paired with An Authorized Autonomous AI for Detecting Diabetic Retinopathy and Diabetic Macular Edema
**Study Purpose**	Diabetic Retinopathy (DR) is the most common cause of blindness in the working population of the United States [1]. Each year, DR leads to more than 50,000 cases of preventable blindness in the United States. An estimated 4.1 million are affected by retinopathy and vision-threatening retinopathy, respectively [2].<br><br>Early detection of DR can prevent vision loss and blindness [3-7]; however, a large portion of individuals with diabetes do not currently undergo routine DR examination [8]. In addition, specific disadvantaged groups have increased prevalence of diabetic retinopathy, where low rates of DR exams are at least partially associated with lack of access to a specialist [9].<br><br>IDx-DR is an autonomous Artificial Intelligence (AI) designed to detect DR and Diabetic Macular Edema (DME) in primary care [10], as well as address the unmet need for DR examinations, lack of access to care, and racial and ethnic disparities in receiving annual diabetic eye exams that have persisted for decades. The Food Drug Administration (FDA) authorized IDx-DR via the De Novo pathway with a Breakthrough Device designation. IDx-DR is currently only indicated with a single fundus camera model, Topcon NW400, for automatically detecting more than mild diabetic retinopathy (mtmDR) in adult subjects diagnosed with diabetes not previously diagnosed with diabetic retinopathy. MtmDR is defined as Early Treatment of Diabetic Retinopathy Study (ETDRS) severity level 35 or higher, and or center-involved, and or clinically significant macular edema. The NW400 is a higher cost camera, making the AI less affordable for those clinics that operate in underserved and under resourced communities.

Digital Diagnostics Confidential. This document is intended for internal use only and may not be disclosed or reproduced in whole, or in part, for external distribution in any form without express written permission of DDx Legal. All hard copies should be checked against the current electronic revision prior to use and destroyed promptly thereafter. All hard copies are considered uncontrolled documents.

	The purpose of this study is to demonstrate substantial equivalence of IDx-DR when paired with a different camera model, the RetinaVue 700 (RV700, Baxter, Skaneateles Falls, NY). Substantial Equivalence will enable Digital Diagnostics to use the RV700 with IDx-DR, which may provide the following benefits:<br>1. More affordable and easier incorporation into primary care workflows, leading to improved racial, ethnic, and geographic access equity for the diabetic eye exam<br>2. Improved provider workflow and efficiency in care delivery
**Objectives**	Primary Objective:<br>To demonstrate the substantial equivalence of IDx-DR using the RV700 to the predicate device (IDx-DR using the NW400) in detecting ETDRS level 35 or higher and / or DME<br><br>Secondary Objectives:<br>1. To determine IDx-DR (using RV700) diagnosability, the percentage of eyes for which a diagnostic result can be provided<br>2. To determine IDx-DR (using RV700) diagnosability, the percentage of subjects for which a diagnostic result can be provided.<br>3. To determine the positive and negative predictive values<br>4. To investigate the effect of cases where no diagnostic result can be provided on sensitivity and specificity using a “worst-case scenario” analysis approach<br><br>Precision Substudy Objective:<br>1. To evaluate the eye-level repeatability and reproducibility of IDx-DR using the RV700<br>2. To evaluate the subject-level repeatability and reproducibility of IDx-DR with the RV700
**Endpoints**	Primary Endpoint:<br>Sensitivity and specificity of IDx-DR using RV700 based on eye-level results against a Level I Prognostic Reference Standard (PRS)<br><br>Secondary Endpoints:

Digital Diagnostics Confidential. This document is intended for internal use only and may not be disclosed or reproduced in whole, or in part, for external distribution in any form without express written permission of DDx Legal. All hard copies should be checked against the current electronic revision prior to use and destroyed promptly thereafter. All hard copies are considered uncontrolled documents.

	1. Sensitivity and specificity of IDx-DR using RV700 based on subject-level results when compared to the Wisconsin Fundus Photograph Reading Center Level I Prognostic Reference Standard (PRS)<br>2. Sensitivity and specificity of IDx-DR using RV700 based on eye-level results when compared to the Wisconsin Fundus Photograph Reading Center Level II Standard grading of the RV700 images<br>3. Sensitivity and specificity of IDx-DR using RV700 based on subject-level results when compared to the Wisconsin Fundus Photograph Reading Center Level II Standard grading of the RV700 images<br>4. Percent of total cases where a diagnostic result can be provided by IDx-DR with the RV700<br>5. Positive Predictive Value (PPV) and Negative Predictive Value (NPV) of IDx-DR with the RV700<br>6. Sensitivity and specificity of IDx-DR as if all PRS-determined positive cases with no diagnostic result provided were false negatives and all PRS-determined negative cases with no diagnostic result were false positives, i.e., a "worst-case scenario" of the impact of "Exam Quality Insufficient" results<br><br>Precision Substudy Endpoints:<br>1. Eye-level DDx algorithm score mixed model estimates of the operator, camera, sequence, dilation, and FPRC effects<br>2. Average positive agreement (APA) in intra- and inter-configurations of RV700 cameras/operators<br>3. Average negative agreement (ANA) in intra- and inter-configurations of RV700 cameras/operators<br>4. Average ungradable agreement (AUA) in intra- and inter-configurations of RV700 cameras/operators<br>5. Overall agreement<br>6. Subject-level DDx algorithm mixed model estimates of the operator, camera, sequence, dilation, and FPRC effects.
**Study procedures**	• Informed Consent and Screening<br>• Diagnostic Performance Study: Fundus Imaging Procedure using IDx-DR with RV700 camera<br>• Precision substudy: repetitions of Fundus Imaging Procedure using combinations of multiple operators using IDx-DR with multiple RV700 cameras (n = 36 subjects)

Digital Diagnostics Confidential. This document is intended for internal use only and may not be disclosed or reproduced in whole, or in part, for external distribution in any form without express written permission of DDx Legal. All hard copies should be checked against the current electronic revision prior to use and destroyed promptly thereafter. All hard copies are considered uncontrolled documents.

**Protocol Number**	DXSDR006
**Study Sponsor**	Digital Diagnostics, Inc.<br>210 5th Street, Suite 103 .<br>Coralville, IA 52241<br>(319) 248-5620
**Study Type**	Prospective diagnostic performance and precision study of a diagnostic medical device.
**Study Product**	IDx-DR is a software device intended for use by healthcare providers to automatically detect mtmDR in adults 22 years and older who have not been previously diagnosed with a retinal disease.<br>IDx-DR analyzes retinal images and provides one of three device outputs: "Diabetic Retinopathy detected: ETDRS level 35 and higher and/or macular edema", "No Diabetic Retinopathy detected: ETDRS level 20 and lower and no macular edema", or "Exam quality insufficient".<br>The RV700 camera will be paired with IDx-DR and evaluated in this study.
**Study Location**	United States
**Planned # of Sites**	At least 2 primary care sites
**Subject Population**	Adults, 22 years or older, living with diabetes, that have not been previously diagnosed with retinal disease.
**Sample Size**	A minimum of 200 eyes with mtmDR and 140 eyes without mtmDR will be enrolled in the main diagnostic performance study, and, of those subjects, 36 will also be enrolled in the precision substudy.
**Subject Duration**	Participation in the main study, the diagnostic performance study, will require two to three hours per subject to complete the RV700 imaging and the imaging required by the retinal reading center. Participation in the precision substudy will require approximately five hours per subject to complete the set of nine RV700 imaging procedures.

Digital Diagnostics Confidential. This document is intended for internal use only and may not be disclosed or reproduced in whole, or in part, for external distribution in any form without express written permission of DDx Legal. All hard copies should be checked against the current electronic revision prior to use and destroyed promptly thereafter. All hard copies are considered uncontrolled documents.

**Study Duration**	The duration of the study is projected to be 5-6 months from enrollment opening to completion of data analysis.
**Study Design**	This is a prospective, multicenter, non-randomized study.
**Subject Eligibility Criteria**	**Subject Inclusion Criteria:**<br>1. 22 years of age or older<br>2. Documented diagnosis of diabetes mellitus, as per any of the following:<br>a. Having met the criteria established by either the World Health Organization (WHO) or the American Diabetes Association (ADA)<br>b. Hemoglobin A1c (HbA1c) $\geq$ 6.5%<br>c. Fasting Plasma Glucose (FPG) $\geq$ 126 mg/dL (7.0 mmol/L)<br>d. Oral Glucose Tolerance Test (OGTT) with two-hour plasma glucose (2-hr PG) $\geq$ 200 mg/dL (11.1 mmol/L), using the equivalent of an oral 75 g anhydrous glucose dose dissolved in water<br>e. Symptoms of hyperglycemia or hyperglycemic crisis with a random plasma glucose (RPG) $\geq$ 200 mg/dL (11.1 mmol/L)<br>3. Ability to understand and the willingness to sign a written informed consent document<br><br>Precision Substudy Additional Inclusion Criteria:<br>1. Enrolled in main study (Diagnostic performance study)<br>2. Received a diagnostic result in the main study<br><br>**Subject Exclusion Criteria:**<br>1. Currently participating in an interventional eye study<br>2. Has a known allergy to or contraindication for the use of Tropicamide 1% or other mydriatic eye drops<br>3. Has a condition that, in the opinion of a licensed clinical team member or investigator, would preclude participation in the study (e.g., unstable medical status including blood pressure or glycemic control, microphthalmia or previous enucleation)<br>4. Pregnancy<br>5. Self-report of visual symptoms including persistent vision loss or blurred vision that cannot be corrected (e.g., with eyeglasses) or floaters<br>6. History of laser treatment of the retina, injections into either eye, or any history of retinal surgery

Digital Diagnostics Confidential. This document is intended for internal use only and may not be disclosed or reproduced in whole, or in part, for external distribution in any form without express written permission of DDx Legal. All hard copies should be checked against the current electronic revision prior to use and destroyed promptly thereafter. All hard copies are considered uncontrolled documents.

	7. Previous confirmed diagnosis of a retinal disease (e.g., macular edema, severe non-proliferative retinopathy, proliferative retinopathy, radiation retinopathy, or retinal vein occlusion)<br>8. Any condition that is contraindicated for the use of the RV700<br>9. Contraindication for imaging by devices used in the study due to any of the following:<br>a. Subject is hypersensitive to light<br>b. Subject recently underwent photodynamic therapy (PDT)<br>c. Subject is taking medication that causes photosensitivity

1.2. Diagnostic Performance Study Workflow

Subjects in the main study, the "Diagnostic Performance Study", will be enrolled at primary care sites and go through two imaging procedures. The first procedure is the RV700 Imaging & AI Procedure, where they will have each retina imaged with an RV700 camera by an operator with no previous fundus imaging experience. The images will be passed to IDx-DR for interpretation and a diagnostic result, as visualized in Section 1.2.2.

After the RV700 Imaging and AI Procedure are complete, subjects will advance to the second imaging procedure to establish the PRS. The 4Widefield Stereo & Macular Spectral Optical Coherence Tomography will be performed by a Fundus Photography Reading Center certified ophthalmic photographer from a qualified reading center. The certified photographer will use an FDA-cleared camera system capable of 4widefield (4W) stereo color fundus photography as well as macular Spectral Optical Coherence Tomography (OCT) capability to perform dilated 4W and macular OCT imaging for each eye. Following the capture of the 4W and OCT images, the subject's participation in the Diagnostic Performance main study will be complete.

All 4W & OCT, & RV700 images will be transferred to the qualified reading center and graded by three retinal experts who will independently grade each image per eye for the PRS, according to the ETDRS severity level, based on the 4W images, including the presence of Clinically Significant Diabetic Macular Edema (CSDME) as well as the presence of Center-Involved DME (CI-DME) from the OCT images. The RV700 will be graded the same way according to the International Clinical Diabetic Retinopathy (ICDR) severity level. The accuracy of IDx-DR using the RV700 camera will be determined in terms of sensitivity and specificity. The PRS (4W & OCT) and ICDR (RV700) will be determined for each eye and aggregated to ETDRS level 20 or less and no DME)" or "ETDRS level 35 or more and/or DME)".

Digital Diagnostics Confidential. This document is intended for internal use only and may not be disclosed or reproduced in whole, or in part, for external distribution in any form without express written permission of DDx Legal. All hard copies should be checked against the current electronic revision prior to use and destroyed promptly thereafter. All hard copies are considered uncontrolled documents.

1.2.1. Diagnostic Performance Study Overall Schema

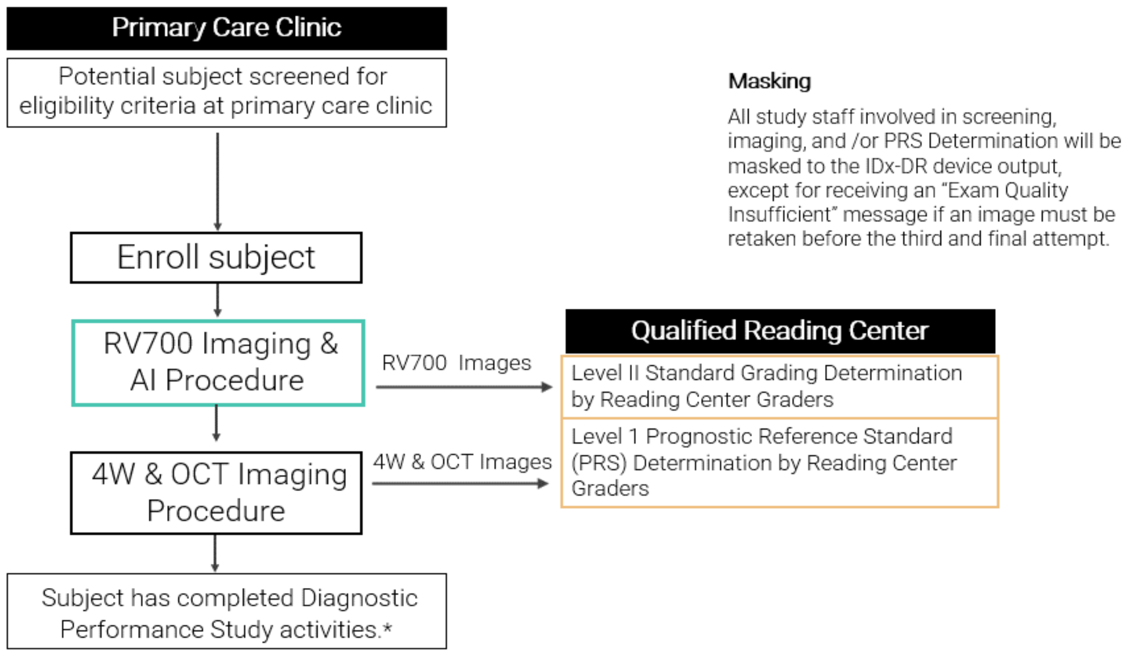

*Select subjects may be invited to subsequently participate in the Precision Substudy.

For more details on the RV700 Imaging & AI Procedure, see Section 1.2.2.

Digital Diagnostics Confidential. This document is intended for internal use only and may not be disclosed or reproduced in whole, or in part, for external distribution in any form without express written permission of DDx Legal. All hard copies should be checked against the current electronic revision prior to use and destroyed promptly thereafter. All hard copies are considered uncontrolled documents.

1.2.2 Diagnostic Performance Study's RV700 Imaging & AI Procedure

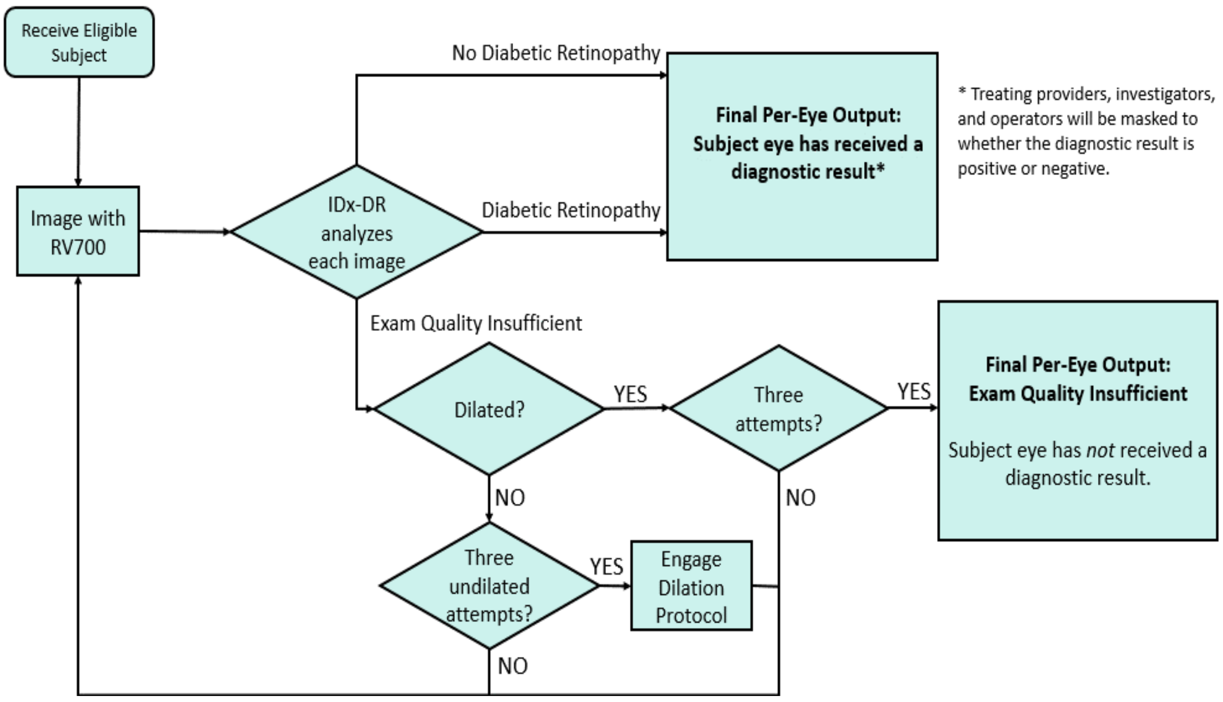

NOTE: Once both eyes of the subject have received a final per-eye output, the subject will then have completed the Diagnostic Performance Study. Digital Diagnostics will be masked to all per-eye outputs.

Digital Diagnostics Confidential. This document is intended for internal use only and may not be disclosed or reproduced in whole, or in part, for external distribution in any form without express written permission of DDx Legal. All hard copies should be checked against the current electronic revision prior to use and destroyed promptly thereafter. All hard copies are considered uncontrolled documents.

1.2.3 Diagnostic Performance Study Schedule of Activities

	Primary Care Sites	
**Evaluations**	**Pre-Study**	**Study Visit**
Medical record review	X	
Informed consent		X
Eligibility assessment		X
Demographics		X
Medical history		X
RV700 Imaging & AI Procedure		X
4W & OCT Imaging Procedure		X
AE evaluation		X
Exit Summary		X

Each enrolled subject will undergo the RV700 Imaging & AI Procedure outlined in Section 1.2.2 using IDx-DR on the RV700 camera.

Subjects who receive diagnostic results for both eyes may be invited to participate in the Precision Substudy.

1.2.4 Diagnostic Performance Study Subject Identification

The subject identifier will be composed of two parts: a two-digit site identifier corresponding to the enrollment site and a unique three-digit subject number separated by a delimiter. For example, the 12th subject enrolled at the third trial site would have a subject identifier "03-012".

1.3. Precision Substudy Workflow

A group of 36 subjects from the Diagnostic Performance Study, 18 of which had at least one eye graded as mtmDR(+) by the PRS, and 18 of which had both eyes graded as mtmDR(-) by the PRS, will be invited to participate in the Precision Substudy. Subjects in the substudy will undergo dilation and then participate in a series of nine IDx-DR exams with various RV700 cameras and operators, in order to test the repeatability and reproducibility of IDx-DR with the RV700. The substudy will test three RV700 camera units and three camera operators with three imaging replicates using a Latin square design. The camera, operator, and replicate imaging combinations are specified in Section 1.3.2. For each of the nine planned evaluations, operators will image the

Digital Diagnostics Confidential. This document is intended for internal use only and may not be disclosed or reproduced in whole, or in part, for external distribution in any form without express written permission of DDx Legal. All hard copies should be checked against the current electronic revision prior to use and destroyed promptly thereafter. All hard copies are considered uncontrolled documents.

subjects' right and left retina, until an image quality result is received per the IDx-DR exam structure outlined in Section 1.3.3.. Subjects will be repositioned between each replicate, within a camera and operator configuration. The inter- and intra-configuration device outputs will then be analyzed to determine mixed effects estimates as well as average positive agreement, average negative agreement, average ungradable agreement, and overall agreement.

1.3.1. Precision Substudy Overall Schema

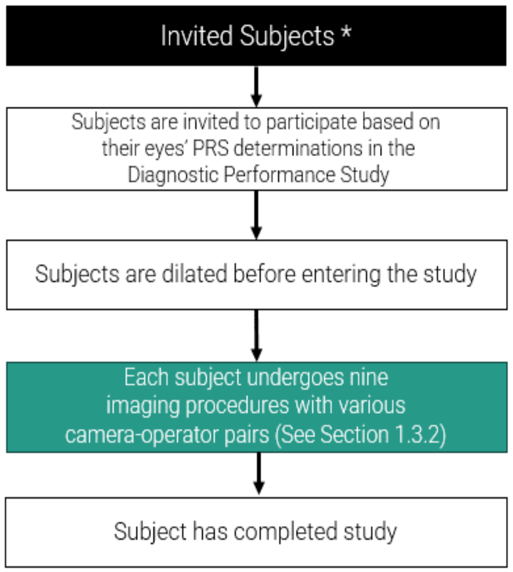

Masking
Operators will be masked from all IDx-DR results and any diagnostic information used to select the subject for the substudy, except for receiving an "Exam Quality Insufficient" message if an image needs to be retaken before the third and final attempt.

***Inviting Subjects from Main Study**
18 subjects who received a mtmDR(+) PRS determination in at least one eye in the Diagnostic Performance Study and 18 subjects who received a mtmDR(-) determination in both eyes will be invited to participate.

Digital Diagnostics Confidential. This document is intended for internal use only and may not be disclosed or reproduced in whole, or in part, for external distribution in any form without express written permission of DDx Legal. All hard copies should be checked against the current electronic revision prior to use and destroyed promptly thereafter. All hard copies are considered uncontrolled documents.

1.3.2. Precision Substudy IDx-DR Exam Procedure

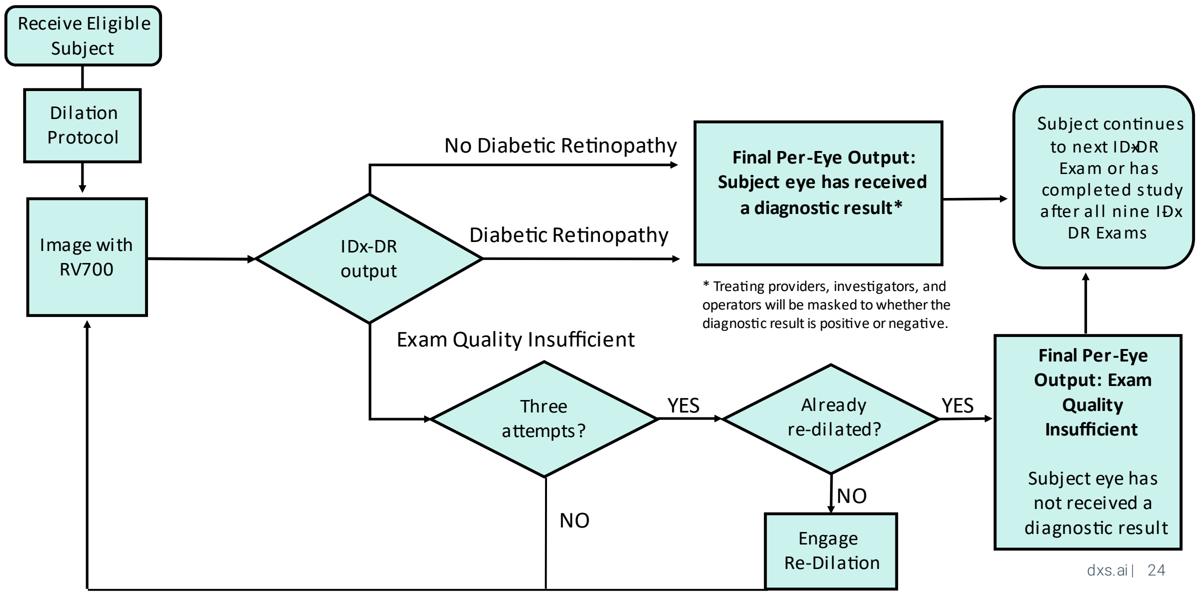

1.3.3 Precision Substudy Camera Operator Configurations

IDx-DR Exam Code	Camera	Operator	Replicate
**A**	A	1	1
**B**		2	1
**C**		2	2
**D**	B	2	1
**E**		3	1
**F**		3	2
**G**	C	1	1
**H**		1	2
**I**		3	1

1.3.4 Precision Substudy Schedule of Activities

Evaluations	Pre-Study	Day of Visit
Eligibility assessment	X	
Dilation		X
Imaging Sequence of IDx-DR Exams		X*
AE evaluation		X

*A series of nine exams with various RV700 cameras and operators are completed per subject as outlined in Sections 1.3.1-1.3.5.

1.3.5 Precision Substudy Subject and IDx-DR Exam Identification

The unique identifier for each exam completed within the Precision Substudy will use the subject's unique identifier from the Diagnostic Performance Study and append the one-character IDx-DR Exam Code (See Section 1.3.4) identifying the operator-camera configuration and repetition number corresponding to that exam. For example, if subject #03-012 from the main study is undergoing the second replicate of Operator 2 and RV700 Camera A, that exam would be identified as 03-012-C.

Digital Diagnostics Confidential. This document is intended for internal use only and may not be disclosed or reproduced in whole, or in part, for external distribution in any form without express written permission of DDx Legal. All hard copies should be checked against the current electronic revision prior to use and destroyed promptly thereafter. All hard copies are considered uncontrolled documents.

2. Introduction

2.1. Background

Diabetic retinopathy (DR) is the most common cause of blindness in the working population of the United States [1]. Each year, DR including diabetic macular edema, leads to more than 50,000 cases of preventable blindness in the United States annually. An estimated 4.1 million, including 899,000 Americans, are affected by retinopathy and vision-threatening retinopathy, respectively [2].

Early detection of DR and DME can prevent vision loss and blindness [3-7]; however, a large portion of individuals with diabetes do not currently undergo routine DR examinations [8]. In addition, specific disadvantaged groups have increased prevalence of diabetic retinopathy, where low rates of DR evaluation are at least partially associated with lack of access to a specialist exam [9].

FDA cleared IDx-DR, an autonomous artificial intelligence (AI) diagnostic system designed to detect DR in primary care [10], as well as address the unmet DR evaluation need, lack of access to care, and racial and ethnic disparity issues that have persisted for decades. IDx-DR is designed, developed, validated, and marketed under a strict ethical framework (16). IDx-DR is currently only indicated with one camera model, Topcon NW400 (Topcon, Pyramus NJ), for the autonomous diagnosis of more than mild diabetic retinopathy (mtmDR), defined as ETDRS severity level 35 or higher, and or macular edema, in adult subjects diagnosed with diabetes not previously diagnosed with diabetic retinopathy.

While many patients have benefitted from the implementation of the IDx-DR system, access is still limited for some populations, such as Federally Qualified Health Centers, which serve low-income communities, and those in nursing homes and mental health institutions, because the IDx-DR is not moveable. It is therefore of the highest importance for health equity, to reach these currently hard-to-serve populations and prevent blindness and visual loss just as well as in other populations. Thus, we selected the highest performing handheld fundus camera (the RV700) and plan to show its safety and accuracy in detecting diabetic retinopathy and macular edema are substantially equivalent to the IDx-DR predicate, with the NW400 camera.

Analyses will be performed at the eye-level and repeated at subject-level for regulatory submission and publication consistent with the de novo study utilizing the Topcon NW400 retinal camera.

Digital Diagnostics Confidential. This document is intended for internal use only and may not be disclosed or reproduced in whole, or in part, for external distribution in any form without express written permission of DDx Legal. All hard copies should be checked against the current electronic revision prior to use and destroyed promptly thereafter. All hard copies are considered uncontrolled documents.

2.2. Study Rationale

The purpose of this study is to demonstrate that IDx-DR with the handheld RetinaVue 700 Retinal Camera (RV700) is substantially equivalent to the predicate device, IDx-DR paired with the Topcon NW400, in terms of sensitivity and specificity. Success will be defined by demonstrating substantial equivalence via non-inferiority.

Showing substantial equivalence may provide the following benefits:

1. Easier incorporation into FQHC primary care workflows, leading to increased access to DR examinations for millions of people living with diabetes
2. Improved provider workflow and efficiency in care delivery

2.3. Benefit/Risk Assessment

The proposed study is expected to introduce only minimal risk to both the camera operators and study subjects. The camera systems being used are commercially available for ocular fundus photography. The Tropicamide 1% eye drops for pupil dilation in this study are approved drug products used in standard practice by ophthalmologists and have demonstrated minimal side effects [11].

The risks associated with using these devices/products in the context of this clinical study are anticipated to be similar to those associated with the use in standard care and are provided below:

- brief discomfort due to light emitted by the fundus camera
- brief discomfort, stinging of the eyes, sensitivity to light, or blurry vision caused by the dilation drops

A serious but rare risk would be an anaphylactic allergic reaction to the Tropicamide 1% eye drops.

As with most diagnostic tools, the principal risks of IDx-DR are those of false positive and false negative results. However, in the context of this clinical study, the IDx-DR results will be masked to operators and treating providers and will not be used to diagnose DR nor guide management or treatment, and therefore do not impact the standard of care or present any increased risk of false positive/negative results.

The benefits associated with using these devices/products in the context of this clinical study are also anticipated to be similar to standard of care along with the addition of the following:

- supporting development of sight saving technologies that may help others with diabetic retinal diseases in the future
- while all subjects enrolled at primary care sites will be referred to ophthalmology according to standard of care, if the prognostic reference standard (PRS) is determined to be mtmDR, the Contract Research Organization (CRO) will notify the study site.

Digital Diagnostics Confidential. This document is intended for internal use only and may not be disclosed or reproduced in whole, or in part, for external distribution in any form without express written permission of DDx Legal. All hard copies should be checked against the current electronic revision prior to use and destroyed promptly thereafter. All hard copies are considered uncontrolled documents.

Considering the risk against benefit assessment, it can be concluded that participation in this study may offer benefit with minimal risk compared to the standard of care procedures.

3. Objectives and Endpoints

3.1. Diagnostic Performance Study

Objectives	Endpoints
**Primary**	
To demonstrate the substantial equivalence of IDx-DR using the RV700 to the predicate device (IDx-DR) in detecting ETDRS level 35 or higher and/or DME	Sensitivity and specificity of IDx-DR using RV700 based on eye-level results against a Level I Prognostic Reference Standard (PRS)
**Secondary**	
1. To determine diagnosability, the percentage of eyes for which a diagnostic result can be provided using IDx-DR with the RV700.<br>2. To determine IDx-DR (using RV700) diagnosability, the percentage of subjects for which a diagnostic result can be provided<br>3. To determine the positive and negative predictive values.<br>4. To investigate the effect of cases where no diagnostic result can be provided on sensitivity and specificity using a "worst case scenario" analysis approach.	1. Sensitivity and specificity of IDx-DR using RV700 based on subject-level results when compared to the Wisconsin Fundus Photograph Reading Center Level I Prognostic Reference Standard (PRS)<br>2. Sensitivity and specificity of IDx-DR using RV700 based on eye-level results when compared to the Wisconsin Fundus Photograph Reading Center Level II Standard grading of the RV700 images<br>3. Sensitivity and specificity of IDx-DR using RV700 based on subject-level results when compared to the Wisconsin Fundus Photograph Reading Center Level II Standard grading of the RV700 images<br>4. Percent of total cases where a diagnostic result can be provided by IDx-DR with the RV700<br>5. Positive Predictive Value (PPV) and Negative Predictive Value (NPV) of IDx-DR with the RV700<br>6. Sensitivity and specificity of IDx-DR as if all PRS-determined positive cases with no diagnostic result provided were false negatives and all PRS-determined negative cases with no diagnostic result were false positives, i.e., a "worst-case scenario" of the impact of "Exam Quality Insufficient" results.

Digital Diagnostics Confidential. This document is intended for internal use only and may not be disclosed or reproduced in whole, or in part, for external distribution in any form without express written permission of DDx Legal. All hard copies should be checked against the current electronic revision prior to use and destroyed promptly thereafter. All hard copies are considered uncontrolled documents.

Precision Substudy	
1. To evaluate the eye-level repeatability and reproducibility of IDx-DR with the RV700.<br>2. To evaluate the subject-level repeatability and reproducibility of IDx-DR with the RV700	1. Eye-level DDx algorithm score mixed model estimates of the operator, camera, sequence, dilation, and FPRC effects<br>2. Average positive agreement (APA) in intra- and inter-configurations of RV700 cameras/operators<br>3. Average negative agreement (ANA) in intra- and inter-configurations of RV700 cameras/operators<br>4. Average ungradable agreement (AUA) in intra- and inter- configurations ofRV700 cameras/operators<br>5. Overall agreement<br><br>6. Subject-level DDx algorithm score mixed model estimates of the operator, camera, sequence, dilation, and FPRC effects

4. Study Design

4.1. Overall Design

This study is a preregistered, multicenter, non-randomized study with primary endpoints to demonstrate the substantial equivalence of IDx-DR paired with the RV700 to the predicate device, IDx-DR paired with the NW400, in detecting mtmDR in subjects not previously diagnosed with retinal disease.

For the main study, the Diagnostic Performance Study, a minimum of 200 eyes with mtmDR and at least 140 eyes without mtmDR will be enrolled at primary care sites. The study population is expected to mirror the spectrum of disease severity encountered at primary care settings, from no DR to proliferative DR (PDR), with a target sample size of at least 200 eyes (~20% of subjects with one or both eyes) with more than mild DR (mtmDR+), which includes ETDRS levels 35-85, and/or clinically significant as well as center-involved macular edema. Within this cohort, at least 40 eyes are expected to have vision-threatening DR (vtDR), defined as ETDRS level 50-85 and/or clinically significant as well as center-involved macular edema. [10]

Enrolled subjects will undergo two fundus imaging procedures. The first procedure, the RV700 Imaging and AI Procedure will involve capturing RV700 images of each eye, in an operator loop that includes quality feedback for the operator which will be passed to IDx-DR for analysis. IDx-DR will provide a diagnostic result of “No diabetic retinopathy detected: ETDRS level 20 and lower and no macular edema” or “Diabetic retinopathy detected: ETDRS level 35 and higher and/or macular edema”, or IDx-DR will determine that no diagnostic result can be detected and output “Exam Quality Insufficient”. In the second procedure, the 4W and OCT Imaging Procedure

Digital Diagnostics Confidential. This document is intended for internal use only and may not be disclosed or reproduced in whole, or in part, for external distribution in any form without express written permission of DDx Legal. All hard copies should be checked against the current electronic revision prior to use and destroyed promptly thereafter. All hard copies are considered uncontrolled documents.

will involve capturing 4W and OCT retinal images by Fundus Photography Reading Center certified photographers, which together with the RV700 images will be transferred to retinal experts at a qualified reading center to determine the prognostic reference standard (PRS) for each eye, respectively ICDR grading for each eye. The study will reach its primary endpoints if the sensitivity and specificity of IDx-DR paired with the RV700 camera are substantially equivalent to those of the predicate device, IDx-DR.

In order to assure an adequate range of DR conditions in the study population sample, and avoid spectrum bias, for sensitivity and specificity analysis, the primary care study population will be enriched to ensure a sufficient range of diabetic retinopathy severity within cases. The enrichment cohort will be targeted using HbA1c levels.

A total of 36 subjects who participated in the Diagnostic Performance Study will be invited to participate in the Precision Substudy. Of these 36 subjects, 18 will have received a PRS determination of mtmDR(+) for at least one eye, and 18 will have received a PRS determination of mtmDR(-) for both eyes. Each participating subject will undergo a series of nine exams with three different RV700 cameras, three different operators, and three replicates to test the reproducibility and repeatability of using IDx-DR with the RV700.

All clinical study sites will have the adequate subject population and availability of space for at least one operator and one RV700 camera. This space needs to be able to be darkened. Two sites will need space for 3 camera operators and 3 RV700 cameras. Operators cannot have previous fundus imaging experience.

The overall study and the substudy analyses will be performed at both the eye-level and the subject-level for regulatory submission and publication consistent with the de novo study utilizing the Topcon NW400 retinal camera.

4.2. Scientific Rationale for Study Design

In order to add an additional fundus camera model to IDx-DR's indications for use, a camera qualification study must be conducted that shows IDx-DR paired with the new camera (RV700) is substantially equivalent to the predicate device, the IDx-DR paired with the NW400. The proposed study is a multicenter, preregistered prospective study design that will measure the sensitivity and specificity of IDx-DR with the RV700. A Level I prognostic reference standard will be determined by retinal experts at a qualified reading center, who will each independently grade the images according to the Early Treatment of Diabetic Retinopathy Study severity scale and the Diabetic Retinopathy Clinical Research Network (DRCR) macular edema severity scale [12] from 4W color, stereoscopic fundus and macular OCT images, and also a Level II reference standard by the same reading centers, reading the RV700 images.

Digital Diagnostics Confidential. This document is intended for internal use only and may not be disclosed or reproduced in whole, or in part, for external distribution in any form without express written permission of DDx Legal. All hard copies should be checked against the current electronic revision prior to use and destroyed promptly thereafter. All hard copies are considered uncontrolled documents.

The subject population is intended to mirror the DR/DME disease spectrum encountered in primary care settings.[10] Primary care sites will be used as enrollment sites for an intent to screen, study design and mimic real-world use at a non-specialist clinic setting.

In order to also study other variables that may impact the use of IDx-DR with the RV700, such as intra-operator and intra-camera variability, a separate substudy focused on precision will be conducted within this study. The Precision Substudy will invite a subset of subjects enrolled in the main study to undergo a series of fundus imaging sessions with various RV700 units and operators.

While subjects in the main Diagnostic Performance Study will only be dilated as needed, all subjects entering the Precision Substudy will undergo dilation. All instances of dilation will use Tropicamide 1%. Repeated imaging causes subject pupil constriction, and therefore, undilated study subjects would require substantial time between imaging sessions for pupil constriction recovery (previously found to be 15 minutes in the IDx-DR pivotal precision substudy). The minimum amount of time required to complete the nine exams within the Precision Substudy protocol with a dilate "as needed" contingency and adequate pupil response recovery would be approximately half a clinic day, making subject enrollment in the afternoon difficult. Therefore, dilation of all subjects prior to the Precision Substudy imaging has been included in the study design.

4.3. IDx-DR: Autonomous AI Device

IDx-DR is an AI diagnostic system that autonomously analyzes images of the retina for signs of diabetic retinopathy. IDx-DR was designed using explicit detectors for biomarkers, which increases robustness and minimizes racial and ethnic biases [13]. The algorithm searches for the same markers of disease that clinicians look for, such as hemorrhages, exudates, neovascularization, and many others.

The American Academy of Ophthalmology (AAO) Preferred Practice Pattern (PPP) (2019) recommends annual monitoring for people with diabetes who have level 20 (ETDRS) diabetic retinopathy or less and are also negative for Diabetic Macular Edema (DME), while those diagnosed with level 35 or higher, or have DME, require more than annual monitoring. According to the AAO PPP, level 35 diabetic retinopathy is the lowest level of diabetic retinopathy that requires more than annual monitoring by an eye care professional. The output of the autonomous AI is thus aligned with the clinical standard for the diagnosis and treatment of diabetic retinopathy.

Digital Diagnostics Confidential. This document is intended for internal use only and may not be disclosed or reproduced in whole, or in part, for external distribution in any form without express written permission of DDx Legal. All hard copies should be checked against the current electronic revision prior to use and destroyed promptly thereafter. All hard copies are considered uncontrolled documents.

IDx-DR provides one of three results:

Exam Results	Diabetic Retinopathy detected, ETDRS level 35 and higher and/or macular edema:<br><br>Refer to an ophthalmologist
Exam Results	No Diabetic Retinopathy detected, ETDRS level 20 and lower and no macular edema:<br><br>Retest in 12 months
Exam Results	Exam Quality Insufficient or Exam Analysis Failed:<br><br>Refer to an ophthalmologist

5. Study Population

Adults with diabetes, 22 years or older, who have not been previously diagnosed with a retinal disease and who meet all eligibility criteria will be eligible for this study. The pivotal trial of IDx-DR included a study sample representative of all 30 million people with diabetes in the US, with corresponding age, racial, and ethnic diversity. Given the focus during IDx-DR development to mitigate racial, ethnic and other biases, its preregistered study design deliberately included hypothesis testing for the presence or absence of such racial, ethnic, age and sex bias in the accuracy of the autonomous AI algorithms [14]. The study showed no effect of race, ethnicity, age, or sex on IDx-DR's sensitivity or specificity [10]. This camera qualification study will enroll a diverse subject population within its smaller sample size but has the advantage of adding to a growing body of evidence, including the pivotal trial's results, demonstrating that IDx-DR is resilient against many forms of bias.

5.1. Clinical Site Selection

The CRO, with the agreement of the Sponsor, will qualify clinical sites according to their interest and ability to participate and to sign an appropriate investigator agreement with the Sponsor, the ability of the investigational site to be reviewed and approved for this study by a qualified IRB, the presence of staffing suitable to conduct protocol-required assessments, the availability to follow this protocol and utilize other written procedures or SOPs regarding the conduct of clinical research, and with input on geographic diversity and suitable patient populations from the study Sponsor.

5.1.1 Clinical Site Selection

The qualification of the sites for the Diagnostic study is the following:

Digital Diagnostics Confidential. This document is intended for internal use only and may not be disclosed or reproduced in whole, or in part, for external distribution in any form without express written permission of DDx Legal. All hard copies should be checked against the current electronic revision prior to use and destroyed promptly thereafter. All hard copies are considered uncontrolled documents.

- Primary care sites with available patient population likely to meet the inclusion/exclusion criteria
- Primary care sites with a broad representative patient population
- Availability of space to have ocular fundus and OCT cameras on site
- Availability staff to train and take fundus photographs per image acquisition guidelines
- Availability of staff who has no previous fundus/retinal image experience
- Internet connection and computer hardware for study system operation (e.g., EDC, analysis dashboard, RVN, etc.).
- Willing to administer tropicamide 1.0% eye drops per protocol requirements
- Previous experience with clinical studies preferred or demonstrated aptitude for conducting study
- Ability to identify a study staff member for WRC certification for the prognostic reference standard imaging using the Maestro system

For the Precision substudy, the Sponsor and CRO acting on behalf of the Sponsor, will select the substudy clinical sites from the sites participating in the Diagnostic study. The substudy sites will be selected based on four criteria: 1) the number of subjects willing to participate in the additional study with particular emphasis on the number of mtmDR participants 2) the willingness of the site and site's staff to participate in the substudy, 3) the ability of the site to store the additional equipment and provide the operators required for the substudy, and 4) the assurance that the site could include an appropriate racial and ethnic diverse population.

For enrichment all sites must be willing to administer HbA1c tests on participants to determine eligibility for enrollment if the subject's medical record lacks adequate documentation to establish the diagnosis of diabetes meeting the enrichment criteria.

5.1.2 Investigator Training

The Sponsor, or designee, will be responsible for training of appropriate clinical site study personnel. prior to study initiation. Training will address topics including the procedure, instrumentation for procedure, adverse events and management of adverse events. Product specific training will include review of product performance information, the study protocol, techniques for the identification of eligible subjects, instructions on data collection and regulatory requirements.

5.2. Screening

A signed Institutional Review Board approved statement of informed consent will be obtained prior to screening activities. The study record will include a statement that written informed consent was obtained and will document the date and time that this written consent was obtained. During the screening period, inclusion/exclusion criteria for study participation will be evaluated. Evaluation of the study subject inclusion/exclusion criteria must be based on the review of medical records and/or documented subject interviews/exams conducted by the Investigator or under the

Digital Diagnostics Confidential. This document is intended for internal use only and may not be disclosed or reproduced in whole, or in part, for external distribution in any form without express written permission of DDx Legal. All hard copies should be checked against the current electronic revision prior to use and destroyed promptly thereafter. All hard copies are considered uncontrolled documents.

direction of the Investigator using qualified study staff (such as nursing staff or research coordinators). There are no screening activities that are outside of standard care practices. Subjects who meet all inclusion criteria and do not meet any exclusion criteria will be eligible to be enrolled. Subjects for the Precision Substudy will be chosen from those enrolled in the main DPS.

5.3. Inclusion Criteria

Diagnostic Performance Study:

1. Diagnostic Performance Study: 22 years of age or older
2. Documented diagnosis of diabetes mellitus, as per any of the following:
    a. Having met the criteria established by either the World Health Organization (WHO) or the American Diabetes Association (ADA)
    b. Hemoglobin A1c (HbA1c) ≥ 6.5%
    c. Fasting Plasma Glucose (FPG) ≥ 126 mg/dL (7.0 mmol/L)
    d. Oral Glucose Tolerance Test (OGTT) with two-hour plasma glucose (2-hr PG) ≥ 200 mg/dL (11.1 mmol/L), using the equivalent of an oral 75 g anhydrous glucose dose dissolved in water
    e. Symptoms of hyperglycemia or hyperglycemic crisis with a random plasma glucose (RPG) ≥ 200 mg/dL (11.1 mmol/L)
3. Ability to understand and the willingness to sign a written informed consent document

Precision Substudy Additional Inclusion Criteria:

1. Enrolled in main study (Diagnostics performance study)
2. Received a diagnostic result in the main study

5.4. Exclusion Criteria

Diagnostic Performance Study:

1. Currently participating in an interventional eye study
2. Has a known allergy to or contraindication for the use of Tropicamide 1% or other mydriatic eye drops
3. Has a condition that, in the opinion of a licensed clinical team member or investigator, would preclude participation in study (e.g., unstable medical status including blood pressure or glycemic control, microphthalmia or previous enucleation)

Digital Diagnostics Confidential. This document is intended for internal use only and may not be disclosed or reproduced in whole, or in part, for external distribution in any form without express written permission of DDx Legal. All hard copies should be checked against the current electronic revision prior to use and destroyed promptly thereafter. All hard copies are considered uncontrolled documents.

4. Pregnancy
5. Self-report of visual symptoms; including persistent vision loss or blurred vision that cannot be corrected (e.g., with eyeglasses), or floaters
6. History of laser treatment of the retina, injections into either eye, or any history of retinal surgery
7. Previous confirmed diagnosis of a retinal disease (e.g., macular edema, severe non-proliferative retinopathy, proliferative retinopathy, radiation retinopathy, or retinal vein occlusion)
8. Any condition that is contraindicated for the use of the RV700
9. Contraindication for imaging by devices used in the study due to any of the following:
    a. Subject is hypersensitive to light
    b. Subject recently underwent photodynamic therapy (PDT)
    c. Subject is taking medication that causes photosensitivity

5.5. Enrollment

Once the statement of Informed Consent is obtained and the screening process is completed, a unique subject identification number will be assigned to subjects. Those who meet all inclusion criteria and do not meet any exclusion criteria will be eligible to be enrolled in the study.

5.6. Screen Failures

Those who do not meet the requirements of the inclusion and exclusion criteria will not be eligible for enrollment in the study. Those who are not eligible for enrollment will be identified as screening failures. The reason for screening failure will be clearly documented in a screening log or master subject log.

5.7. Enrichment Inclusion and Exclusion Criteria

The following inclusion and exclusion criteria are as follows for enrichment in the Diagnostic Performance study. Subjects' eligibility for the study will be based on inclusion and exclusion criteria referenced in section 5.3. and 5.4 above.

5.7.1 Inclusion criteria for enrichment:

1. Diagnosis of diabetes mellitus (as defined above in Section 5.3, except as follows): HbA1c level as defined by statistician during enrichment period
2. Age 22 years or older
3. Understand the study and willingness to sign the informed consent

Digital Diagnostics Confidential. This document is intended for internal use only and may not be disclosed or reproduced in whole, or in part, for external distribution in any form without express written permission of DDx Legal. All hard copies should be checked against the current electronic revision prior to use and destroyed promptly thereafter. All hard copies are considered uncontrolled documents.

5.7.2 Exclusion criteria for enrichment:

1. Persistent vision loss, blurred vision, or floaters
2. Diagnosed with DME, severe NPDR, proliferative retinopathy, radiation retinopathy, or retinal vein occlusion
3. History of laser treatment of the retina or injections into either eye, or any history of retinal surgery
4. Participating in another investigational eye study and actively receiving investigational product for DR or DME at the time of the current study
5. Subject had a condition that, in the opinion of the investigator, precluded participation in the study (e.g., unstable medical status including blood pressure or glycemic control, microphthalmia or previous enucleation)
6. Subject was contraindicated for imaging by fundus imaging system used in the study:
    - Subject was hypersensitive to light.
    - Subject recently underwent PDT.
    - Subject was taking medication that causes photosensitivity

5.8. Subject Discontinuation/Withdrawal from the Study

A subject may withdraw from the study at any time at his/her own request or may be withdrawn at any time at the discretion of the Investigator for safety, behavioral, compliance, or administrative reasons.

If the subject withdraws consent for disclosure of future information, the Sponsor may retain and continue to use any data collected before the withdrawal of consent.

5.9. Off Study/End of Study Definition

5.9.1. Off Study

Subjects will be considered Off Study at the time of withdrawal of full consent, once they have completed the study, or at the End of Study (defined below). Subjects will also be taken Off Study due to any of the following:

- If at any time in the study, dilation is deemed to be contraindicated for the subject.
- If subject is non-compliant with study visits or study procedures.
- Investigator determines that continuing on study is not in the best interest of the subject.

5.9.2. End of Study

Digital Diagnostics Confidential. This document is intended for internal use only and may not be disclosed or reproduced in whole, or in part, for external distribution in any form without express written permission of DDx Legal. All hard copies should be checked against the current electronic revision prior to use and destroyed promptly thereafter. All hard copies are considered uncontrolled documents.

A subject is considered to have completed the Diagnostic Performance Study and, if invited to participate, the Precision Substudy, if they have completed all study activities including the last scheduled procedure shown in the Schedule of Activities in Section 1.2.3 and 1.3.5, respectively.

The End of Study is defined as the date of the last scheduled procedure shown in the Schedule of Activities in Sections 1.2.3 and 1.3.5 for the last subject in either study.

5.10. Discontinuation of Entire Study

This study, including the substudy, may be discontinued for any of the following reasons:

- The study is completed per-protocol.
- Determination of unexpected, significant, and/or unacceptable risk to subjects.
- The Sponsor may terminate the study electively or if required by regulatory decision.

If the study is terminated prematurely, the Sponsor will notify the investigators, IRB(s), and regulatory authorities (if applicable) of the decision and the reason for terminating the study.

6. Devices Used in this Study

IDx-DR is an AI diagnostic system intended for use by healthcare providers to automatically detect more than mild diabetic retinopathy (mtmDR) in adults (22 years of age or older) diagnosed with diabetes who have not been previously diagnosed with diabetic retinopathy.

6.1. Fundus Camera to be Used in this Study

The fundus camera that will be used in this clinical study is the RetinaVue 700 retinal camera (RV700, Baxter, Skaneateles Falls, NY) (study camera). The clinical site operators will follow the procedures for this camera will be provided with training by the Sponsor.

6.2. IDx-DR

The IDx-DR device consists of component parts IDx-DR Client, IDx-Service, and IDx-DR Analysis.

6.2.1. IDx-DR Client

IDx-DR Client is a software application component responsible for assembling and submitting a set of images to IDx-DR Analysis via IDx-Service. In the context of the study, a version of the IDx-DR Client has been developed that submits images captured by the RV700 camera to IDx-Service for processing. This component runs as part of a dedicated study cloud infrastructure and uses all the best practices to securely transmit and store information.

Digital Diagnostics Confidential. This document is intended for internal use only and may not be disclosed or reproduced in whole, or in part, for external distribution in any form without express written permission of DDx Legal. All hard copies should be checked against the current electronic revision prior to use and destroyed promptly thereafter. All hard copies are considered uncontrolled documents.

6.2.2. IDx-Service

IDx-Service comprises a software application that contains a web server front-end that securely validates incoming requests, a database that stores information, a job queuing system, and a logging system that records information about each transaction through IDx-Service. IDx-Service is also primarily responsible for cybersecurity. For this study, customized study software will fulfill the role of IDx-Service to enable Digital Diagnostics to be masked to all image data and results.

6.2.3. IDx-DR Analysis

IDx-DR Analysis is the analysis software that analyzes the subject's images to determine exam quality and the presence/absence of diabetic retinopathy.

6.3. Preparation/Handling/Storage of Devices

The cameras and computer hardware are to be handled and stored safely and properly per the manufacturer's recommendations. A device accountability log will be maintained by the study site and reviewed by the study monitor.

6.4. Measures to Minimize Bias

The Sponsor (DDx) will be masked for this clinical study. The IDx-Client will assemble and submit images to the study analysis system. The analysis system is designed to analyze the subject's images to determine exam quality and the presence/absence of diabetic retinopathy. The data will be housed in a secure server during study and after completion. During the study, necessary maintenance activities, cybersecurity updates, or system access necessary for the continuing operation of the server will be performed by authorized personnel, who will only act upon instruction from an authorized Digital Diagnostics personnel. Prior to enrolling subjects in the clinical trial, Digital Diagnostics is responsible for installation of the final and locked IDx-DR algorithm. Digital Diagnostics will place a copy of the algorithm in escrow (e.g., source code) prior to the start of enrollment for auditing purposes. All activities on the system will be logged and recorded. The study data will be escrowed, as was the original pivotal trial, in other words, there will be a complete independent audit trail of who had access to which study when. Such escrowed study data will be fully masked to Digital Diagnostics Staff. [Abramoff et al, 2021].

Documentation, logging and an auditable record will provide evidence of two important items:
1) Digital Diagnostics does not have direct access to patient-level data during the course of the study, and
2) After the study, patient-level data will continue to be masked to DDx.

This study is a non-randomized, preregistered multicenter study. Subject study identifiers will be assigned by the clinical site at the time of screening. The Diagnostic Performance Study

Digital Diagnostics Confidential. This document is intended for internal use only and may not be disclosed or reproduced in whole, or in part, for external distribution in any form without express written permission of DDx Legal. All hard copies should be checked against the current electronic revision prior to use and destroyed promptly thereafter. All hard copies are considered uncontrolled documents.

subject identifier will be composed of two parts: a two-digit site identifier and a three-digit unique subject number separated by a delimiter. For example: the 3rd trial site enrolling their 12th subject will have a subject identifier of "03-012". The Precision Substudy will make use of these subject identifiers and append a one-character IDx-DR Exam ID separated by a delimiter, corresponding to the specific camera-operator-replicate configuration (see table in Section 1.3.4). For example, if subject 03-012 is invited into the Precision Substudy, assigned to Imaging Sequence 1, then that exam would be identified as "03-012-1".

7. Study Assessments and Procedures

7.1. Screening

Procedures conducted as part of the subject's routine clinical management and obtained before the signing of the Informed Consent Form (ICF) may be utilized for pre-screening or baseline purposes provided it meets the protocol-specified criteria. Screening includes a review of demographics to include age, medical history/current medical conditions, disease history and extent of disease, and prior therapies and/or documented subject interviews/exams. All screening evaluations must be completed and reviewed to confirm that potential subjects meet all eligibility criteria.

The Investigator is obliged to give the subject thorough information about the study and the study-related assessments, and the subject should be given ample time to consider their participation. The Investigator must not start any study-related procedure before the ICF is signed and dated by both subject and the person who conducted the informed consent discussion (Investigator, or a person designated by the Investigator).

The clinical site will maintain a screening log to record details of all subjects screened and to confirm eligibility or record reasons for screening failure, as applicable.

7.2. Diagnostic and Demographic Data

Clinical study staff will review the subject's medical records and/or consult with the subject to collect the following data including but not limited to:

- subject diagnoses of diseases that manifest in the retina, including but not limited to, glaucoma, diabetic retinopathy, age-related macular degeneration, etc.
- history of laser treatment of the retina or retinal surgery (including intraocular injections)
- family history of retinal disease
- information regarding diabetes to include but not limited to:
    - Date of diagnosis or length of time with diabetes
    - Type 1 or Type 2

Digital Diagnostics Confidential. This document is intended for internal use only and may not be disclosed or reproduced in whole, or in part, for external distribution in any form without express written permission of DDx Legal. All hard copies should be checked against the current electronic revision prior to use and destroyed promptly thereafter. All hard copies are considered uncontrolled documents.

- Last HbA1c result
- Date of last diabetic retinopathy eye exam
- Comorbidities/complications of diabetes such as cardiovascular disease, neuropathy, nephropathy, peripheral arterial disease
- Treatments with insulin therapy and/or oral medications

- Demographic data including age, sex, and race/ethnicity

7.2.1. Collection of Race and Ethnicity Data

Race and ethnicity information must be self-reported and may be multiracial. The following choices are offered:

Ethnicity:

- **Hispanic or Latino:** A person of Cuban, Mexican, Puerto Rican, South or Central American, or other Spanish culture or origin, regardless of race.

Race:

- **American Indian or Alaska Native:** A person having origins in any of the original peoples of North and South America (including Central America), and who maintains tribal affiliation or community attachment.
- **Asian:** A person having origins in any of the original peoples of the Far East, Southeast Asia, or the Indian subcontinent, including, for example, Cambodia, China, India, Japan, Korea, Malaysia, Pakistan, the Philippine Islands, Thailand, and Vietnam.
- **Black or African American:** A person having origins in any of the black racial groups of Africa.
- **Native Hawaiian or Other Pacific Islander:** A person having origins in any of the original peoples of Hawaii, Guam, Samoa, or other Pacific Islands.
- **White:** A person having origins in any of the original peoples of Europe, the Middle East, or North Africa.

Once the subject has provided informed consent, been deemed eligible after screening, and provided all requested medical history, the subject will proceed with study activities.

7.3. Representation Across Disease Spectrum

To ensure representation across disease spectrum and avoid spectrum bias, a minimum of 200 eyes with mtmDR and 140 eyes without mtmDR will be enrolled at primary care sites for the Diagnostics Performance Study. The study population is expected to mirror the spectrum of disease severity encountered at primary care settings. Of the 200 mtmDR+ eyes, at least 40 eyes are expected to have vtDR.

Digital Diagnostics Confidential. This document is intended for internal use only and may not be disclosed or reproduced in whole, or in part, for external distribution in any form without express written permission of DDx Legal. All hard copies should be checked against the current electronic revision prior to use and destroyed promptly thereafter. All hard copies are considered uncontrolled documents.

7.4. Enrichment Cohort

In order to assure an adequate range of DR conditions in the study population sample, and avoid spectrum bias, for sensitivity and specificity analysis, the primary care study population may be enriched following the pre-specified formal evaluations outlined in the Data Transfer Plan, to ensure a sufficient range of diabetic retinopathy severity within cases. The enrichment cohort will be targeted using HbA1c levels, if needed, and will draw from the same pool of intent to screen subjects as the non-enriched subjects. To avoid excessive enrollment in any one stratum, the totals will be monitored weekly. Participant enrichment will be targeted based on elevated HbA1c, factors which have been shown to be correlated with higher rates of mtmDR among patients with diabetes. No cap will be placed on enriched participants since such participants will be prospectively recruited. The goal will be to recruit enough subjects to satisfy each stratum. If enrollment patterns are not on track with the study's target population after periodic reviews, enrollment at all sites will be adjusted monthly by modifying the HbA1c. Adjustments from this level may be instituted after a periodic review of the stratum enrollments reflecting the number of participants with diabetic retinopathy or diabetic macular edema. Study sites will be notified by the CRO if there is to be a change in the HbA1c thresholds. Once the total number of cases has been projected in alignment with statistical power requirements, then the final adjustments to participant enrollment criteria will be performed.

Monitoring of strata will be performed by the study statistician (without access to IDx-DR results); only available HbA1c, along with FPRC categorization into mtmDR and vtDR, will be visible for each subject included to date. The study statistician will notify the CRO whether updated HbA1c thresholds are needed. Again, once the strata targets for mtmDR and no/mild DR can be projected, the HbA1c thresholds will be locked.

7.5. PRS Diagnostic Performance Study RV700 Imaging & AI Procedure

All subjects who are enrolled in the Diagnostic Performance Study will proceed to the RV700 Imaging & AI Procedure as outlined in Section 1.2.1. and 1.2.2. An operator, who has been provided with training by the Sponsor but who has no previous fundus imaging experience, will take images of the subject's left and right retina with an RV700 camera. The images will be immediately passed to IDx-DR for analysis. The operator will be masked to IDx-DR's diagnostic results but will be notified if the submitted images are insufficient for one or both eyes. If the images are not sufficient, the operator will receive an output of "Exam Quality Insufficient" and need to repeat the imaging process for that eye(s). If the operator receives three successive results

Digital Diagnostics Confidential. This document is intended for internal use only and may not be disclosed or reproduced in whole, or in part, for external distribution in any form without express written permission of DDx Legal. All hard copies should be checked against the current electronic revision prior to use and destroyed promptly thereafter. All hard copies are considered uncontrolled documents.

of “Exam Quality Insufficient” for either eye, the subject’s eyes will be dilated. The subject will first be asked to remove contact lenses if they are wearing contacts. Tropicamide 1% eye drops will be administered by qualified site staff under medical supervision. The subject will then be asked to wait at least 30 minutes or until the pupils are dilated to at least 5 mm. The operator will then attempt to image and submit images again. If after a further three attempts a diagnostic result is still not received, no further imaging attempts will be executed, PRS and the IDx-DR output for that/those eye(s) will be recorded as “Exam Quality Insufficient”. After the RV700 Imaging and AI Procedure is complete, subjects will advance to the PRS imaging protocol, the 4Widefield stereo & macular OCT Imaging Procedure performed by a FPRC certified ophthalmic photographer. The FPRC certified photographer will use a different, FDA-cleared camera system for widefield stereo color fundus photography as well as macular Spectral Optical Coherence Tomography (OCT) capability to perform dilated 4W four-field fundus photography and macular (OCT) imaging for each eye. Following the capture of the 4W and OCT images, the subject’s participation in the Diagnostic Performance main study will be complete.

After completion of the Diagnostic Performance Study, RV700, 4W and OCT images from each eye will be passed to retinal grading experts at a qualified reading center, who will independently determine the prognostic reference standard (PRS) for each eye from the 4W & OCT images, and ICDR grading from the RV700 images, and will be masked to each other and the IDx-DR diagnostic output. If the image is insufficient in any way, the ophthalmologist can record a determination of “ungradable”.

7.6 Precision Substudy IDx-DR Exam Procedure

Select subjects will be invited back for the Precision Substudy based on the PRS grading of their Diagnostic Performance Study images. Eighteen (18) of the Precision Substudy subjects will have received a grade of mtmDR(+) in at least one eye, and eighteen (18) will have received a grade of mtmDR(-) in both eyes. These subjects will undergo the IDx-DR Exam Procedure as outlined in Section 1.3.1-1.3.4. First, Tropicamide 1% eye drops will be administered by qualified site staff under medical supervision. The subject will then be asked to wait at least 30 minutes or until the pupils are dilated to at least 5 mm. The fully dilated subject will then undergo nine IDx-DR exams. The substudy will test three RV700 cameras, and three camera operators with three pre-specified replicates, such that each subject will complete nine pairs of imaging exams. The camera, operator and replicate combinations across subjects are outlined below:

Exam A: RV700 A + Operator #1 (Replicate 1)
Exam B: RV700 A + Operator #2 (Replicate 1)
Exam C: RV700 A + Operator #2 (Replicate 2)
Exam D: RV700 B + Operator #2 (Replicate 1)
Exam E: RV700 B + Operator #3 (Replicate 1)
Exam F: RV700 B + Operator #3 (Replicate 2)

Digital Diagnostics Confidential. This document is intended for internal use only and may not be disclosed or reproduced in whole, or in part, for external distribution in any form without express written permission of DDx Legal. All hard copies should be checked against the current electronic revision prior to use and destroyed promptly thereafter. All hard copies are considered uncontrolled documents.

Exam G: RV700 C + Operator #1 (Replicate 1)
Exam H: RV700 C + Operator #1 (Replicate 2)
Exam I: RV700 C + Operator #3 (Replicate 1)

Operators will be instructed to reposition subjects between each imaging replicate. Operators will also be instructed to give subjects at least five minutes between exams to have time for adequate pupil recovery. If within a given exam the subject is unable to receive a diagnostic result in one or both eyes, the procedure will be repeated. If after three attempts a diagnostic result is not received and the subject has not already been re-dilated, Tropicamide 1% eye drops will be re-administered by qualified site staff under medical supervision. If after another three attempts a diagnostic result is not received, the per-eye output will be recorded as "Exam Quality Insufficient", and the subject will continue to the next IDx-DR Exam until all nine exams are completed. In no circumstance will a subject receive Tropicamide more than twice in the substudy. This workflow is shown visually in Section 1.3.3.

The camera operators for this procedure will be operators with no previous retinal imaging experience. The Sponsor will ensure the site has three operators who are able to complete an IDx-DR training program on the RV700. Operators will be masked from all IDx-DR results and any diagnostic information used to select the subject for the substudy, except in the case where no diagnostic result can be provided, and the device output is "Exam Quality Insufficient". In that case, the operator will be notified to attempt the exam again, re-dilate after three failed attempts, or after an additional three failed attempts, consider the device output for that eye in that exam to be "Exam Quality Insufficient", and proceed with the next exam.

The Precision Substudy IDx-DR Exam Procedure may take more than three hours to complete. If the subject's pupils constrict to less than 5mm, Tropicamide 1% eye drops will be re-administered by qualified site staff under medical supervision.

8. Criteria for Evaluation

8.1. Primary Efficacy Endpoint

The co-primary endpoints are the sensitivity and specificity of IDx-DR with the RV700 detecting more than mild DR (mtmDR) based on eye-level results in subjects recruited from primary care clinics when compared to a prognostic reference standard.

For secondary efficacy endpoints, the following will be analyzed:

1) Sensitivity and specificity of IDx-DR using RV700 based on subject-level results against a Level I Prognostic Reference Standard (PRS)
2) Sensitivity and specificity of IDx-DR using the RV700cbased on eye-level results when compared to the Wisconsin Fundus Photograph Reading Center Level II Standard grading of the RV700 images

Digital Diagnostics Confidential. This document is intended for internal use only and may not be disclosed or reproduced in whole, or in part, for external distribution in any form without express written permission of DDx Legal. All hard copies should be checked against the current electronic revision prior to use and destroyed promptly thereafter. All hard copies are considered uncontrolled documents.

3) Sensitivity and specificity of IDx-DR using RV700 based on subject-level results when compared to the Wisconsin Fundus Photograph Reading Center Level II Standard grading of the RV700 images
4) Percent of total cases where a diagnostic result can be provided by IDx-DR with the RV700
5) Positive Predictive Value (PPV) and Negative Predictive Value (NPV) of IDx-DR with the RV700
6) Sensitivity and specificity of IDx-DR as if all PRS-determined positive cases with no diagnostic result provided were false negatives and all PRS-determined negative cases with no diagnostic results were false positives, i.e., a "worst case scenario" of the impact of "Exam Quality Insufficient" results.

For the Precision Substudy, the following will be analyzed:

1) Eye-level DDx algorithm score mixed model estimates of the operator, camera, sequence, dilation, and replicate effects
2) Average Positive Agreement (APA) in intra- and inter- configurations of RV700 cameras/operators
3) Average Negative Agreement (ANA) in intra- and inter- configurations of RV700 cameras/operators
4) Average Ungradable Agreement (AUA) in intra- and inter- configurations of RV700 cameras/operators
5) Overall agreement
6) Subject-level DDx algorithm mixed model estimates of the operator, camera, sequence, dilation, and replicate effect.

Section 9 has further details on the primary, secondary, and Precision Substudy endpoint analyses.

8.2. Safety Assessments

The proposed study is expected to introduce only minimal risk to both the camera operators and subjects because the RV700 camera is commercially available for ocular fundus photography and Tropicamide 1% eye drops for pupil dilation are an FDA-approved drug product used in standard care and have been demonstrated to have minimal side effects [11].

8.2.1. Safety Monitoring

Ongoing safety monitoring will be performed by the CRO on behalf of the Sponsor. The CRO will review reported AEs received from the clinical study sites on a regular basis and report them promptly to the Sponsor. Investigators and the Sponsor will be notified if unreported AEs are observed in study records by the monitors during routine or for cause monitoring activities. The

Digital Diagnostics Confidential. This document is intended for internal use only and may not be disclosed or reproduced in whole, or in part, for external distribution in any form without express written permission of DDx Legal. All hard copies should be checked against the current electronic revision prior to use and destroyed promptly thereafter. All hard copies are considered uncontrolled documents.

Sponsor will review, document, and report (if applicable) adverse events (AEs), including Adverse Device Effects (ADEs) or Unanticipated Adverse Device Effects (UADEs), submitted by clinical investigators. Events considered serious and unexpected by the site staff or the CRO will be immediately investigated by the Sponsor to determine if the event qualifies for expedited reporting to regulatory authorities.

<u>Adverse Event</u>

An adverse event (AE) is defined as any untoward medical occurrence, unintended disease or injury, or untoward clinical signs whether or not related to the investigational medical device. This includes events related to the medical device and events related to the procedures involved.

<u>Serious Adverse Event</u>

A Serious Adverse Event (SAE) is defined as an AE that:

- Led to death;
- Led to serious deterioration in the health of the subject, users, or other persons as defined by one or more of the following that resulted in:
    - a life-threatening illness or injury, or
    - a permanent impairment of a body structure or a body function, or
    - hospital admission (>24 hours) or prolongation of existing hospitalization, or
    - medical or surgical intervention to prevent permanent life-threatening illness or injury, or permanent impairment to a body structure or a body function;
- Led to fetal distress, fetal death, or a congenital anomaly or birth defect

Note: Planned hospitalization for a pre-existing condition, or a procedure required by the protocol, without serious deterioration in health, is not considered a serious adverse event.

The Sponsor will be responsible for coordinating the expedited reporting of serious and unexpected AEs to regulatory authorities within the required timeframes once the determination for reporting has been made.

8.2.2. Methods and Timing for Assessing, Recording, and Analyzing Safety Parameters

Safety will be evaluated during the time the subject is in the study, which will include from the time of consent through the time of exit. Safety will be assessed by frequency and incidence of ADEs and UADEs.

Digital Diagnostics Confidential. This document is intended for internal use only and may not be disclosed or reproduced in whole, or in part, for external distribution in any form without express written permission of DDx Legal. All hard copies should be checked against the current electronic revision prior to use and destroyed promptly thereafter. All hard copies are considered uncontrolled documents.

Because this is considered a device study, it is consistent to use the following definitions when assessing safety issues:

- **Adverse Device Effect (ADE)**, which means an adverse event related to the use of an investigational medical device.
- **Unanticipated Adverse Device Effect (UADE)**, which means any serious adverse effect on health or safety, or any life-threatening problem or death caused by or associated with its use that had not been previously identified in nature, severity, or degree of incidence.

8.2.3. Identification of Events and Timeframe for Reporting

For the purpose of this study, ADEs and UADEs will only be recorded for the duration of the subject's involvement in the study. Many subjects in this study may have pre-existing medical conditions; however, those pre-existing conditions will not be considered as ADE/UADEs, unless use of the study device elicits a new ADE/UADE or worsens an existing condition in terms of frequency or intensity during or immediately after the use of the study device. All reportable events as defined above, determined to be an ADE/UADE will be recorded in the source documents and entered in the electronic case report form (e-CRF). The Investigator will provide the date of onset and resolution, intensity, frequency, action(s) taken, changes in study device application, relationship to study device, and outcome.

8.2.4. Follow-up of Adverse Device Effects

ADEs that occur on the day of the visit will be followed up to adequately evaluate the subject's safety or until the event stabilizes. When the event resolves, a resolution date will be documented on the case report form. All UADEs will be followed until resolution or until the subject is medically stable. All other events that cannot be resolved by 30 days after the last study contact will be considered resolved by convention and entered in the Electronic Data Capture (EDC) system.

8.2.5. Guidelines for Assessing Intensity of an Adverse Device Effect

The Investigator should use the following definitions when assessing intensity of an adverse event:

- MILD: Subject is aware of symptoms or has minor findings but tolerates them well, and no or minimal intervention required
- MODERATE: Subject experiences enough symptoms or findings to require intervention
- SEVERE: Subject experiences symptoms or findings that require significant medical intervention

Digital Diagnostics Confidential. This document is intended for internal use only and may not be disclosed or reproduced in whole, or in part, for external distribution in any form without express written permission of DDx Legal. All hard copies should be checked against the current electronic revision prior to use and destroyed promptly thereafter. All hard copies are considered uncontrolled documents.

8.2.6. Guidelines for Determining Causality

The Investigator will use the following question when assessing causality of an ADE/UADE to study device, where an affirmative answer designates the event as a suspected ADE: Is there a reasonable possibility that the device or its use caused the event? ‘‘Reasonable possibility’’ means that there is evidence to suggest a causal relationship between use of the device and the ADE.

8.2.7. Reporting Procedures

All recordable ADEs will be entered into the EDC within 7 days of identification. Recordable UADEs will be reported within 3 working days of identification by telephone to the Sponsor at (319) 248-5620 and entered into the EDC as soon as possible thereafter.

8.2.8. Anticipated Adverse Device Effects

Due to the non-invasive nature of the devices, there are no anticipated adverse device effects directly related to IDx-DR or RV700.

The proposed study is expected to introduce only minimal risk to both the camera operators and subjects because the RV700 cameras are commercially available for ocular fundus photography and Tropicamide 1% eye drops for pupil dilation are an approved drug product used in standard care and have been demonstrated to have minimal side effects [11].

The risks associated with using these devices/products in the context of this clinical study are anticipated to be similar to those associated with the use in standard care and are provided below:

- brief discomfort due to light emitted by the fundus cameras
- brief discomfort, stinging of the eyes, sensitivity to light, or blurry vision caused by the dilation drops

8.2.9. Unanticipated Adverse Device Effects

Investigators must report any UADE to the Sponsor immediately. The Sponsor is obligated to investigate UADE reports promptly to determine reporting requirements of the event and assess if the event warrants further action by the Sponsor.

8.2.10. Protocol Deviations

A deviation is defined as an event that did not occur according to the protocol requirements. Protocol deviations will be documented on the Protocol Deviation CRF. Deviations are reported per the governing IRB requirements.

Digital Diagnostics Confidential. This document is intended for internal use only and may not be disclosed or reproduced in whole, or in part, for external distribution in any form without express written permission of DDx Legal. All hard copies should be checked against the current electronic revision prior to use and destroyed promptly thereafter. All hard copies are considered uncontrolled documents.

8.2.11. Regulatory Reporting

Any event that may be considered a UADE related to the RV700 paired with the IDx-DR device, and therefore may require reporting based on federal regulations, will be forwarded to the Sponsor for investigation. After assessing reporting requirements of the event, the Sponsor or its representative will submit reports to the FDA and other regulatory agencies as necessary and will inform the investigators of such regulatory reports. Site investigators must submit safety reports as required by their IRB. Documentation of the submission and receipt by the IRB must be retained for each safety report.

8.2.12. Type and Duration of Follow-up of Subjects after ADEs

ADEs/UADEs will be followed until resolved or considered stable. Adverse events will be followed by the Investigator or a qualified member of the study team. The Investigator or a qualified study team member may review the subject's medical record, contact the subject by phone, or contact the subject's primary care physician for follow-up. Subject safety data will be reviewed on an ongoing basis by the Sponsor.

9. Statistical Considerations

9.1. Hypothesis testing

In this study a minimum of 200 eyes with mtmDR and at least 140 eyes without mtmDR will be enrolled in the main diagnostic performance study. The device performance will be evaluated per eye. The primary endpoints will be sensitivity and specificity of IDx-DR paired with the RV700 in detecting mtmDR based on eye-level results when compared to a PRS by three retinal experts. Intent-to-treat (ITT) analyses are planned; supportive analyses are planned (see Statistical Analysis Plan [SAP] table shells).

As this study is intended to support an extension of the IDx-DR labeling to validate a new camera model for use with IDx-DR, the performance is expected to be substantially equivalent to the predicate device. Substantial equivalence will be assessed as non-inferiority since higher than expected performance is a favorable outcome. The non-inferiority targets will be set based on Sponsor expectations for eye-level sensitivity and specificity. Published results of this trial and NW400 subject-level estimates are summarized in Supplementary Table 7

Supplemental Table 7: Eye-level diagnostic design parameters and pivotal study subject-level performance

Digital Diagnostics Confidential. This document is intended for internal use only and may not be disclosed or reproduced in whole, or in part, for external distribution in any form without express written permission of DDx Legal. All hard copies should be checked against the current electronic revision prior to use and destroyed promptly thereafter. All hard copies are considered uncontrolled documents.

	Eye-level Null Hypothesis p_0	Subject-level Point Estimate (two-sided 95% CI)
Sensitivity	75.0%	87.2% (81.8%-91.2%)
Specificity	77.5%	90.7% (88.3%-92.7%)

The study population is expected to mirror the spectrum of disease severity encountered at primary care settings, from no DR to proliferative DR (PDR), with a target sample size of at least 200 eyes (~20% of subject sample size) with more than mild DR (mtmDR+), which includes ETDRS levels 35-85, and/or clinically significant as well as center-involved macular edema as subgroup analyses. The study population is also expected to mirror the racial and ethnic distribution of the US diabetes population. Within this cohort, at least 40 eyes are expected to have vision threatening DR (vtDR), defined as ETDRS level 50-85 and/or clinically significant as well as center-involved macular edema. In addition, the same analyses will be performed at the subject-level for regulatory submission and publication, but the subject-level analyses will not have formal study hypotheses.

9.1.1 Sample Size, Precision, and Power

Minimum sample sizes of n=200 mtmDR+ eyes and 140 mtmDR- eyes will be required. The predicate device, IDx-DR with the NW400, pivotal trial pre-specified null hypotheses for sensitivity and specificity of 75% and 77.5%, respectively; these represent pre-defined non-inferiority margins. The alternative hypotheses were 85.0% for sensitivity and 82.5% for specificity in support of a non-inferiority design. These lower thresholds for performance will be ruled out with one-sided 97.5% confidence bounds.

As this study is intended to support an extension of the IDx-DR labeling to validate a new camera for use with IDx-DR, the performance is expected to be substantially equivalent to the predicate device at the subject-level.

This study will validate the use of a new camera with the same diagnostic algorithm as currently on the market. The proposed sample size maintains a one-sided 97.5% confidence interval that exceeds the predicate device pivotal trial null hypotheses, i.e., a lower confidence limit >75% for sensitivity and >77.5% for specificity which reflects a 5% non-inferiority offset for sensitivity and a 2.5% non-inferiority offset for specificity (**Supplementary Table 8** columns 1-4). These hypothesis tests were designed specifically to reflect clinically meaningful eye-level outcomes; substantial equivalence is interpreted as non-inferiority (**Supplementary Table 8** columns 1-4).

Supplementary Table 8: Power and Significance Threshold Calculations Summary (minimum sample sizes)

Digital Diagnostics Confidential. This document is intended for internal use only and may not be disclosed or reproduced in whole, or in part, for external distribution in any form without express written permission of DDx Legal. All hard copies should be checked against the current electronic revision prior to use and destroyed promptly thereafter. All hard copies are considered uncontrolled documents.

	Sponsor Criteria (Non-inferiority)			
	Sensitivity		Specificity	
	Power	N-I	Power	N-I
	**1**	2	3	4
Test significance level, alpha	0.025	0.025	0.025	0.025
1 or 2 sided test?	1	1	1	1
Null hypothesis %, p_0	75%	75%	77.5%	77.5%
Alternative hypothesis %, p_A	83.5%	81.2%	87.1%	84.6%
Power (%)	80	NA	80	NA
N	200	200	140	140

9.2. Demographics

Age will be summarized by eye and by subject using descriptive statistics, and sex, ethnicity, and race will be summarized using counts and percentages. Summaries will be done by diagnostic stratum and overall. Sequential enrollment versus enrichment enrollment will be summarized.

9.3. Endpoints

9.3.1. Primary Endpoint

The primary objective of this study is to estimate the sensitivity and specificity of IDx-DR with the RV700 in detecting mtmDR based on eye-level results when compared to a prognostic reference standard. For each eye from each subject enrolled, IDx-DR results will be obtained as positive for mtmDR (hereafter, positive), negative for mtmDR (negative), or exam quality insufficient/failed (ungradable). Similarly, results of positive, negative, or ungradable will be determined by the majority vote of retinal experts at a reading center as the prognostic reference standard. The unit of analysis will be the eye, and the superiority targets will be set based on expecting the point estimate for sensitivity and specificity to be the same as the observed performance of IDx-DR with the NW400 camera measured in the pivotal trial.

9.3.2. Secondary Endpoints

Secondary endpoints include the following:

- Sensitivity and specificity of IDx-DR using RV700 based on subject-level results against a Level I Prognostic Reference Standard (PRS)

Digital Diagnostics Confidential. This document is intended for internal use only and may not be disclosed or reproduced in whole, or in part, for external distribution in any form without express written permission of DDx Legal. All hard copies should be checked against the current electronic revision prior to use and destroyed promptly thereafter. All hard copies are considered uncontrolled documents.

- Sensitivity and specificity of IDx-DR using RV700 based on eye-level results when compared to the Wisconsin Fundus Photograph Reading Center Level II Standard grading of the RV700 images
- Sensitivity and specificity of IDx-DR using RV700 based on subject-level results when compared to the Wisconsin Fundus Photograph Reading Center Level II Standard grading of the RV700 images
- Percent of total cases where a diagnostic result can be provided by IDx-DR with the RV700
- Positive Predictive Value (PPV) and Negative Predictive Value (NPV) of IDx-DR with the RV700
- Sensitivity and specificity of IDx-DR categorizing all PRS-determined positive cases with no diagnostic result as false negatives and all PRS-determined negative cases with no diagnostic result as false positives, i.e., a "worst case scenario" analysis of the impact of "Exam Quality Insufficient" results.

9.3.3. Definitions for Primary and Secondary Endpoint Calculations

	Prognostic reference standard			
	Positive	Negative	Ungradable	Total
IDx-DR Result				
Positive	PP	PN	PU	DP
Negative	NP	NN	NU	DN
Ungradable	UP	UN	UU	DU
Total	TP	TN	TU	N

Endpoints:

$$\text{Sensitivity} = \frac{PP}{PP+NP}$$

$$\text{Specificity} = \frac{NN}{PN+NN}$$

$$\text{Positive Predictive Value} = \frac{PP}{PP+PN}$$

$$\text{Negative Predictive Value} = \frac{NN}{NP+NN}$$

Digital Diagnostics Confidential. This document is intended for internal use only and may not be disclosed or reproduced in whole, or in part, for external distribution in any form without express written permission of DDx Legal. All hard copies should be checked against the current electronic revision prior to use and destroyed promptly thereafter. All hard copies are considered uncontrolled documents.

$$\text{Diagnosibility} = \frac{PP+PN+NP+NN}{TP+TN}$$

Endpoints considering ungradable eyes:

$$\text{Sensitivity}_{+u} = \frac{PP}{PP+NP+UP}$$

$$\text{Specificity}_{+u} = \frac{NN}{PN+NN+UN}$$

$$\text{PPV}_{+u} = \frac{PP}{PP+PN+PU}$$

$$\text{NPV}_{+u} = \frac{NN}{NP+NN+NU}$$

9.3.4. Analyses

A cross tabulation of IDx-DR results (positive, negative, ungradable) with the results from the reference standard (positive, negative, ungradable) will be presented for the overall sample. The point estimate for each test characteristic will be presented along with the counts of eyes (numerator and denominator) that contribute to the calculation. One-sided 97.5% confidence intervals will be computed by the cluster bootstrap approach to account for correlation introduced by taking measurements from both eyes of each subjectif the confidence interval is not estimable using the cluster bootstrap approach due to small sample sizes or boundary values (e.g., a test characteristic of 100%), then the exact binomial confidence interval will be presented instead. Primary endpoints will be reported in stratified analyses by eye, age, sex, and race/ethnicity. Should enrichment be implemented, further stratification of all endpoints will be presented by cohort: sequential enrollment or enrichment.

Relevant data analyses will be repeated at the subject-level for regulatory submission and publication consistent with the de novo study utilizing the Topcon NW400 retinal camera.

Data analysis will be performed using SAS (V9.3 or later).

9.4. Precision Substudy

A repeatability and reproducibility substudy will be performed including three cameras, three operators, and three replicates. The unit of analysis will be the eye (two eyes included per subject). Each of 36 subjects, 50% positive for mtmDR in at least one eye, will have a total of 9 results on each eye (see Section 9.4.1). Select subjects will be invited for the Precision Substudy based on the PRS grading of their Diagnostic Performance Study images. Eighteen (18) of the Precision Substudy subjects will have received a grade of mtmDR(+) in at least one eye, and eighteen (18) will have received a grade of mtmDR(-) in both eyes. Subjects will be fully dilated and then undergo nine IDx-DR exams. The substudy will test three RV700 cameras and three operators with three replicates using a Latin Square design.

Digital Diagnostics Confidential. This document is intended for internal use only and may not be disclosed or reproduced in whole, or in part, for external distribution in any form without express written permission of DDx Legal. All hard copies should be checked against the current electronic revision prior to use and destroyed promptly thereafter. All hard copies are considered uncontrolled documents.

The camera, operator, and replicate combinations across subjects are specified below:

Exam A: RV700 A + Operator #1 (Replicate 1)
Exam B: RV700 A + Operator #2 (Replicate 1)
Exam C: RV700 A + Operator #2 (Replicate 2)
Exam D: RV700 B + Operator #2 (Replicate 1)
Exam E: RV700 B + Operator #3 (Replicate 1)
Exam F: RV700 B + Operator #3 (Replicate 2)
Exam G: RV700 C + Operator #1 (Replicate 1)
Exam H: RV700 C + Operator #1 (Replicate 2)
Exam I: RV700 C + Operator #3 (Replicate 1)

The Latin Squares design follows where the X represents an observation and R represents a replicate in order to estimate camera effect, operator effect, and reproducibility:

	Operator 1	Operator 2	Operator 3
Camera A	X	X, R	-
Camera B	-	X	X, R
Camera C	X, R	-	X

Operators will reposition subjects between each imaging replicate. The order of the data collection will be recorded in the case report form.

9.4.1. Imaging Sequence for Precision Study

Camera	Operator	Replicate
A	1	1
	1	2
	1	3
B	2	1
	2	2
	2	3

Digital Diagnostics Confidential. This document is intended for internal use only and may not be disclosed or reproduced in whole, or in part, for external distribution in any form without express written permission of DDx Legal. All hard copies should be checked against the current electronic revision prior to use and destroyed promptly thereafter. All hard copies are considered uncontrolled documents.

C	3	1
	3	2
	3	3
Total gradings / eye		9
Subjects per setting		36

Endpoints for each analysis include mixed effects estimates of operator, camera, and replicate effects as well as overall agreement, average positive agreement, average negative agreement, and average ungradable agreement. Reproducibility, or inter-operator variability, will also be assessed based on agreement between three camera-operator pairs for a given subject (see Section 9.4.2). One-sided 97.5% confidence intervals for each endpoint will be computed using the clustered bootstrap approach. [12]

Data analysis will be performed using SAS (V9.3 or later).

Relevant data analyses will be repeated at the subject-level for regulatory submission and publication, consistent with the de novo study utilizing the Topcon NW400 retinal camera.

9.4.2. 3 by 3 Cross-Tabulation for Agreement Statistics

Cross-Tabulation for Agreement Statistics

	R2 Positive	R2 Negative	R2 Ungradable
R1 Positive	PP	PN	PU
R1 Negative	NP	NN	NU
R1 Ungradable	UP	UN	UU

R1 and R2 represent replicates 1 and 2

Formulas:

$$\text{Overall agreement (OA)} = \frac{PP+NN+UU}{Total}$$

$$\text{Average positive agreement (APA)} = \frac{2PP}{(PP+NP+UP)+(PP+PN+PU)}$$

$$\text{Average negative agreement (ANA)} = \frac{2NN}{(PN+NN+UN)+(NP+NN+NU)}$$

$$\text{Average ungradable agreement (AUA)} = \frac{2UU}{(PU+NU+UU)+(UP+UN+UU)}$$

Digital Diagnostics Confidential. This document is intended for internal use only and may not be disclosed or reproduced in whole, or in part, for external distribution in any form without express written permission of DDx Legal. All hard copies should be checked against the current electronic revision prior to use and destroyed promptly thereafter. All hard copies are considered uncontrolled documents.

10. Regulatory, Ethical, and Study Oversight Considerations

Ethical Considerations

This study will be performed in accordance with the protocol and all applicable local regulatory requirements. The study will be conducted in accordance with the ethical principles that have their origins in the Declaration of Helsinki.

Institutional Review Board

It is the responsibility of the Investigator to obtain the approval of the Institutional Review Board (IRB) before the start of the study. A copy of the approval letter will be supplied to the Sponsor, along with a roster of IRB members. During the course of the study, the Investigator or designee will provide timely and accurate reports to the IRB on the progress of the study at appropriate intervals (not to exceed 1 year) and at the completion of the study. Investigator or designee will notify the IRB of SAEs and/or UADE, or other important safety findings. The study protocol ICF information sheet advertisements (if any), and amendments (if any) will be approved by the IRB at each study site in conformance with CFR, Title 21, Part 56.

Subject Information and Consent

Before the start of any study-related procedures are undertaken, the Investigator or designee must obtain written, informed consent from each study subject in accordance with US federal regulations (21 CFR §50) and the ICH document "Guidance for Industry – E6 Good Clinical Practice: Consolidated Guidance". Informed consent will be obtained by discussing with the subject the purpose of the study, the risks and benefits, the study procedures, and any other information relevant to the subject.

The Investigator or designee must explain to the subject that for purposes of evaluating the study results, that subject's private health information obtained during the study may be shared with the Sponsor, regulatory agencies, and IRBs, before enrolling that subject into the study. It is the Investigator's (or designee's) responsibility to obtain permission to use private health information per the Health Information Portability and Accountability Act (HIPAA) from each subject, or if appropriate, the subject's legal representative.

The subject or his/her legal representative will document his/her informed consent by signing the current version of the written, IRB-approved ICF. The person who conducted the informed consent discussion with the subject and/or subject's legal representative must also sign the ICF. The subject is given a fully executed copy of the ICF bearing all appropriate signatures, and the original must be maintained in the clinical study master files at the site.

Digital Diagnostics Confidential. This document is intended for internal use only and may not be disclosed or reproduced in whole, or in part, for external distribution in any form without express written permission of DDx Legal. All hard copies should be checked against the current electronic revision prior to use and destroyed promptly thereafter. All hard copies are considered uncontrolled documents.

The Investigator, or designee, is responsible for the content of the ICF, but the original and any updated versions must be approved by the Sponsor prior to submission to the IRB. The ICF should also include any additional information required by local laws relating to institutional review. All active subjects participating on the protocol must be re-consented each time the ICF is updated and re-approved by the IRB.

Investigator and Clinical Sites

The Principal Investigator (PI) at each study site is the individual responsible for ensuring that the investigation is conducted according to the signed Investigator's statement, the protocol, and guidelines at their site. The PI at each clinical site will be responsible for the management of the study, which will include but not be limited to oversight of other designated study Investigators and study staff conducting any activities related to the study, maintenance of the study file and subject records, correspondence with the IRB, and completion of the electronic case report forms (eCRFs).

Data Monitoring

Before the first subject is enrolled in the study, a Sponsor or CRO representative will meet with the Investigator and clinical site study staff to review the procedures for conducting the study and to train the staff on recording the data on the eCRFs using the EDC system. The Sponsor representative may periodically monitor the progress of the study by conducting on–site visits o virtual visits. The Sponsor representative will also be able to review data remotely, which may warrant more frequent communication with the Investigators and clinical site study staff. The Investigator will make available to the Sponsor representative the source documents, the signed consent forms, all other study-related documents, and the computer(s) that access the eCRFs. The Investigator or designee will be responsible for reviewing eCRFs, resolving data queries generated by the Sponsor or CRO, providing missing or corrected data, approving all changes to the data, and endorsing the subject data within the EDC system.

Data Management and Transfer

Masking and privacy are essential data transfer and management elements during the study. The IDx-DR Client will securely transfer images captured from subjects to an analysis system that provides exam quality feedback results, housed, and maintained in a secure server. The study details on the data transfer and management responsibilities can be found in the Data Transfer Plan (DTP as well as the enrichment approach for the study. The DTP serves to designate data transfer and management responsibilities during the study's setup, during enrollment, and after the study to ensure data integrity and security, as referenced in Section 6.4. Additionally, the DTP describes the study data management and transfer responsibilities for the CRO, WRC, and the biostatistician while ensuring Digital Diagnostics remains masked in the study The DTP also provides the details on the study's enrichment approach along with the personnel involved in the execution of the enrichment process.

Digital Diagnostics Confidential. This document is intended for internal use only and may not be disclosed or reproduced in whole, or in part, for external distribution in any form without express written permission of DDx Legal. All hard copies should be checked against the current electronic revision prior to use and destroyed promptly thereafter. All hard copies are considered uncontrolled documents.

Data Recording and Documentation
Data collection will involve the use of the EDC system. In addition to periodic monitoring occurring within the system by CRO personnel, programmatic edit checks will be used to review the data for completeness, logic, and adherence to study protocol. As a result of this monitoring and these checks, queries may be issued to the clinical study site and answered by that study site.

Data will be stored and evaluated in such a way as to guarantee subject confidentiality in accordance with the legal stipulations applying to confidentiality of data. Study records (e.g., copies of eCRFs, regulatory documents, etc.) will be retained at the study site, along with adequate source documentation. All study records must be available for inspection by the Sponsor, and its authorized representatives.

Retention and Review of Records
The PI must maintain the documentation relating to this study. If the Sponsor, CRO, or regulatory authority wishes to review any documentation relating to the study, the PI must permit access to such records. If the PI retires, relocates, or for other reasons withdraws from the responsibility of keeping the study records, custody must be transferred to a suitable alternate custodian employee of the study site or to a suitably qualified and responsible third party. The Sponsor must be notified in writing of the name and address of the new custodian before such transfer is made.

No study records shall be destroyed without notifying and giving the Sponsor the opportunity to arrange long-term storage for such study records or to authorize in writing the destruction of records after the required retention period.

Reports Required from Investigators to Sponsor

Type of Report	Prepared by PI for	Notification Time Frame
UADE	Sponsor, IRB	As soon as possible, but no later than 3 working days of knowledge
Death	Sponsor,<br>IRB if required	As soon as possible, but no later than 3 working days of knowledge

Digital Diagnostics Confidential. This document is intended for internal use only and may not be disclosed or reproduced in whole, or in part, for external distribution in any form without express written permission of DDx Legal. All hard copies should be checked against the current electronic revision prior to use and destroyed promptly thereafter. All hard copies are considered uncontrolled documents.

SAE	Sponsor,<br><br>IRB if required	Within 3 working days of knowledge<br><br>Per IRB requirement
Device malfunction with clinical sequelae	Sponsor<br><br>IRB, if required	Within 3 working days via written communication. Return the device to Sponsor as requested.
Serious protocol deviations (e.g., ICF not obtained, to protect the life or physical well-being of a subject in an emergency)	Sponsor<br><br>IRB, if required	Within 5 working days of knowledge<br><br>Per IRB requirement
Withdrawal of IRB approval	Sponsor	Within 5 working days of knowledge
Progress report	Sponsor, IRB	As required by IRB
Final report	Sponsor, IRB	Within 3 months of study completion or termination
Note: Each IRB may require more stringent reporting requirements than those listed in this table		

Subject Confidentiality
All subject records will only be identifiable by a unique subject identification number. Subjects' names or identifying information other than the data as specified for the eCRF collection are not to be transmitted to the Sponsor. The PI will keep a master subject list on which the subject number and full name of each subject are listed.

Study Audits and Inspections
The study may be evaluated by the Sponsor and/or designees and government inspectors who must be allowed access to eCRFs, source documents, and other study files. Sponsor audit reports will be kept confidential. The Investigator should promptly notify Sponsor of any audits scheduled by any regulatory authorities, and promptly forward copies of audit reports.

Study Termination

Digital Diagnostics Confidential. This document is intended for internal use only and may not be disclosed or reproduced in whole, or in part, for external distribution in any form without express written permission of DDx Legal. All hard copies should be checked against the current electronic revision prior to use and destroyed promptly thereafter. All hard copies are considered uncontrolled documents.

The Sponsor reserves the right to terminate the study in its entirety or at a specific study site at any time.

Registration and Publication

The Sponsor will pre-register this study on the clinical trials registry available at www.clinicaltrials.gov. Publication of the results by the Investigators and/or Sponsor will be subject to the signed contractual agreement(s) executed prior to initiation of the study.

Digital Diagnostics Confidential. This document is intended for internal use only and may not be disclosed or reproduced in whole, or in part, for external distribution in any form without express written permission of DDx Legal. All hard copies should be checked against the current electronic revision prior to use and destroyed promptly thereafter. All hard copies are considered uncontrolled documents.

11. References

1. Klonoff, D.C. and Schwartz, D.M., An economic analysis of interventions for diabetes. Diabetes Care, 2000. 23(3): p. 390-404.
2. CDC. Vision Health Initiative. 2015 [cited 2015 August 4, 2015]; Available from: http://www.cdc.gov/visionhealth/basic_information/eye_disorders.htm#a5.
3. Bressler, N.M., et al., Underuse of the health care system by persons with diabetes mellitus and diabetic macular edema in the United States. JAMA Ophthalmol, 2014. 132(2): p. 168-73.
4. Early photocoagulation for diabetic retinopathy. ETDRS report number 9. Early Treatment Diabetic Retinopathy Study Research Group. Ophthalmology, 1991. 98(5 Suppl): p. 766-785.
5. Photocoagulation for diabetic macular edema. Early Treatment Diabetic Retinopathy Study report number 1. Early Treatment Diabetic Retinopathy Study research group. Arch. Ophthalmol., 1985. 103(12): p. 1796-1806.
6. Diabetic Retinopathy Study, G., Photocoagulation treatment of proliferative diabetic retinopathy: clinical application of DRS findings: DRS report 8. Ophthalmology, 1981. 88: p. 583-600.
7. Diabetic Retinopathy Study, G., Indications for photocoagulation treatment of diabetic retinopathy: DRS report 14. Int Ophthalmol Clin, 1987. 27: p. 239-253.
8. Benoit, S.R., et al., Eye Care Utilization Among Insured People with Diabetes in the U.S., 2010–2014. Diabetes Care, 2019 March. 42(3): p. 427-433.
9. Lee, D.J., et al., Dilated eye examination screening guideline compliance among patients with diabetes without a diabetic retinopathy diagnosis: the role of geographic access. BMJ Open Diabetes Res Care, 2014. 2(1): p. e000031.
10. Abràmoff, M.D., Lavin, P.T., Birch, M. et al., Pivotal trial of an autonomous AI-based diagnostic system for detection of diabetic retinopathy in primary care offices. npj Digital Med, 2018. 39(1).
11. Inan, U.U., Ozturk, F., and Ermis, S.S., Pharmacologic pupil dilation in diabetic patients. Retina, 2003. 23(2): p. 254-6.
12. Wilkinson CP, Ferris FL 3rd, Klein RE, et al., Proposed international clinical diabetic retinopathy and diabetic macular edema disease severity scales. Ophthalmology, 2003. 110(9):1677-1682.
13. Shah, Abhay et al., Validation of automated screening for referable diabetic retinopathy with an autonomous diagnostic artificial intelligence system in a Spanish population. Journal of diabetes science and technology, 2021. 15(3): p. 655-663.

Digital Diagnostics Confidential. This document is intended for internal use only and may not be disclosed or reproduced in whole, or in part, for external distribution in any form without express written permission of DDx Legal. All hard copies should be checked against the current electronic revision prior to use and destroyed promptly thereafter. All hard copies are considered uncontrolled documents.

14. Abràmoff, Michael D et al., Lessons Learned About Autonomous AI: Finding a Safe, Efficacious, and Ethical Path Through the Development Process. American journal of ophthalmology, 2020. 214: p. 134-142.
15. Ying, GS et al., Calculating sensitivity, specificity, and predictive values for correlated eye data. Investigative ophthalmology & visual science, 2020. 61(11): p. 29.
16. Abramoff, M.D., B. Cunningham, B. Patel, M.B. Eydelman, T. Leng, T. Sakamoto, R. M. Wolf, A.K. Manrai, J.M. Ko, and M.F. Chiang. "Foundational Considerations for Artificial Intelligence ", Ophthalmology (2021). https://doi.org/10.1016./j.ophtha.2021.08-023.

Digital Diagnostics Confidential. This document is intended for internal use only and may not be disclosed or reproduced in whole, or in part, for external distribution in any form without express written permission of DDx Legal. All hard copies should be checked against the current electronic revision prior to use and destroyed promptly thereafter. All hard copies are considered uncontrolled documents.

12. Revision History

Revision Level	Description of Change(s)
1.0	Initial Release
2.0	• Modified the number of eyes (+mtmDR) and (- mtmDR) throughout due to an increase in sample size, where applicable<br>• Updated the statistical section of the protocol<br>• Updated Figure 1.2.1 (Diagnostic Performance Study Overall Schema)<br>• Added a secondary endpoint to address grading of RV700 images by WRC via ICDR Level II<br>• Included enrichment strategy if applicable<br>• Added language to specify the following:<br>➢ Clinical site selection criteria for Diagnostic Performance Study<br>➢ Site selection criteria for the Precision Substudy<br>➢ Definition of SAE<br>➢ Data Transfer Plan<br>➢ Device accountability<br>➢ A table on reporting timelines from Investigator to Sponsor
2.1	• Administrative changes<br>• Clarification of the screening process<br>• Added confirmatory language regarding the implementation of enrichment
3.0	• Modification to Diagnostic Performance Study secondary objectives and corresponding efficacy endpoints and planned analysis<br>• Modification to Precision Substudy endpoints and corresponding substudy schema and planned analysis<br>• Clarification to enrichment strategy evaluations<br>• Included minor clarifications to subject exclusion criteria: (5) and (7)<br>• Administrative updates where applicable

Digital Diagnostics Confidential. This document is intended for internal use only and may not be disclosed or reproduced in whole, or in part, for external distribution in any form without express written permission of DDx Legal. All hard copies should be checked against the current electronic revision prior to use and destroyed promptly thereafter. All hard copies are considered uncontrolled documents.

Digital Diagnostics Confidential. This document is intended for internal use only and may not be disclosed or reproduced in whole, or in part, for external distribution in any form without express written permission of DDx Legal. All hard copies should be checked against the current electronic revision prior to use and destroyed promptly thereafter. All hard copies are considered uncontrolled documents.

Section & Topic	No	Item	Reported on page #
**TITLE OR ABSTRACT**			
	**1**	Identification as a study of diagnostic accuracy using at least one measure of accuracy (such as sensitivity, specificity, predictive values, or AUC)	2,3
**ABSTRACT**			
	**2**	Structured summary of study design, methods, results, and conclusions (for specific guidance, see STARD for Abstracts)	2
**INTRODUCTION**			
	**3**	Scientific and clinical background, including the intended use and clinical role of the index test	3
	**4**	Study objectives and hypotheses	
**METHODS**			
Study design	**5**	Whether data collection was planned before the index test and reference standard were performed (prospective study) or after (retrospective study)	8
Participants	**6**	Eligibility criteria	9
	**7**	On what basis potentially eligible participants were identified (such as symptoms, results from previous tests, inclusion in registry)	9
	**8**	Where and when potentially eligible participants were identified (setting, location and dates)	9
	**9**	Whether participants formed a consecutive, random or convenience series	10
Test methods	**10a**	Index test, in sufficient detail to allow replication	11
	**10b**	Reference standard, in sufficient detail to allow replication	11
	**11**	Rationale for choosing the reference standard (if alternatives exist)	11
	**12a**	Definition of and rationale for test positivity cut-offs or result categories of the index test, distinguishing pre-specified from exploratory	12
	**12b**	Definition of and rationale for test positivity cut-offs or result categories of the reference standard, distinguishing pre-specified from exploratory	12
	**13a**	Whether clinical information and reference standard results were available to the performers/readers of the index test	12
	**13b**	Whether clinical information and index test results were available to the assessors of the reference standard	12
Analysis	**14**	Methods for estimating or comparing measures of diagnostic accuracy	13
	**15**	How indeterminate index test or reference standard results were handled	13
	**16**	How missing data on the index test and reference standard were handled	13
	**17**	Any analyses of variability in diagnostic accuracy, distinguishing pre-specified from exploratory	13
	**18**	Intended sample size and how it was determined	13
**RESULTS**			
Participants	**19**	Flow of participants, using a diagram	24
	**20**	Baseline demographic and clinical characteristics of participants	3
	**21a**	Distribution of severity of disease in those with the target condition	3
	**21b**	Distribution of alternative diagnoses in those without the target condition	3
	**22**	Time interval and any clinical interventions between index test and reference standard	11
Test results	**23**	Cross tabulation of the index test results (or their distribution) by the results of the reference standard	4
	**24**	Estimates of diagnostic accuracy and their precision (such as 95% confidence intervals)	4
	**25**	Any adverse events from performing the index test or the reference standard	4
**DISCUSSION**			
	**26**	Study limitations, including sources of potential bias, statistical uncertainty, and generalisability	4
	**27**	Implications for practice, including the intended use and clinical role of the index test	4
**OTHER INFORMATION**			
	**28**	Registration number and name of registry	2
	**29**	Where the full study protocol can be accessed	15
	**30**	Sources of funding and other support; role of funders	15

CC BY SA

STARD 2015

AIM

STARD stands for “Standards for Reporting Diagnostic accuracy studies”. This list of items was developed to contribute to the completeness and transparency of reporting of diagnostic accuracy studies. Authors can use the list to write informative study reports. Editors and peer-reviewers can use it to evaluate whether the information has been included in manuscripts submitted for publication.

EXPLANATION

A **diagnostic accuracy study** evaluates the ability of one or more medical tests to correctly classify study participants as having a **target condition.** This can be a disease, a disease stage, response or benefit from therapy, or an event or condition in the future. A medical test can be an imaging procedure, a laboratory test, elements from history and physical examination, a combination of these, or any other method for collecting information about the current health status of a patient.

The test whose accuracy is evaluated is called **index test.** A study can evaluate the accuracy of one or more index tests. Evaluating the ability of a medical test to correctly classify patients is typically done by comparing the distribution of the index test results with those of the **reference standard**. The reference standard is the best available method for establishing the presence or absence of the target condition. An accuracy study can rely on one or more reference standards.

If test results are categorized as either positive or negative, the cross tabulation of the index test results against those of the reference standard can be used to estimate the **sensitivity** of the index test (the proportion of participants *with* the target condition who have a positive index test), and its **specificity** (the proportion *without* the target condition who have a negative index test). From this cross tabulation (sometimes referred to as the contingency or “2x2” table), several other accuracy statistics can be estimated, such as the positive and negative **predictive values** of the test. Confidence intervals around estimates of accuracy can then be calculated to quantify the statistical **precision** of the measurements.

If the index test results can take more than two values, categorization of test results as positive or negative requires a **test positivity cut-off**. When multiple such cut-offs can be defined, authors can report a receiver operating characteristic (ROC) curve which graphically represents the combination of sensitivity and specificity for each possible test positivity cut-off. The **area under the ROC curve** informs in a single numerical value about the overall diagnostic accuracy of the index test.

The **intended use** of a medical test can be diagnosis, screening, staging, monitoring, surveillance, prediction or prognosis. The **clinical role** of a test explains its position relative to existing tests in the clinical pathway. A replacement test, for example, replaces an existing test. A triage test is used before an existing test; an add-on test is used after an existing test.

Besides diagnostic accuracy, several other outcomes and statistics may be relevant in the evaluation of medical tests. Medical tests can also be used to classify patients for purposes other than diagnosis, such as staging or prognosis. The STARD list was not explicitly developed for these other outcomes, statistics, and study types, although most STARD items would still apply.

DEVELOPMENT

This STARD list was released in 2015. The 30 items were identified by an international expert group of methodologists, researchers, and editors. The guiding principle in the development of STARD was to select items that, when reported, would help readers to judge the potential for bias in the study, to appraise the applicability of the study findings and the validity of conclusions and recommendations. The list represents an update of the first version, which was published in 2003.

More information can be found on [http://www.equator-network.org/reporting-guidelines/stard.](http://www.equator-network.org/reporting-guidelines/stard)

CC BY SA
